# YBX1/CD36 positive feedback loop-mediated lipid accumulation drives metabolic dysfunction-associated steatotic liver disease

**DOI:** 10.7150/ijbs.105798

**Published:** 2025-02-18

**Authors:** Qingqing Zhang, Fei Li, Qichao Ge, Yihui Wang, Zhenyang Shen, Yuecheng Guo, Junjun Wang, Hanjing Zhangdi, Jingyi Lu, Jiaqi Gao, Guangwen Chen, Qidi Zhang, Xingpeng Wang, Hui Dong, Lungen Lu

**Affiliations:** 1 Department of Gastroenterology, Shanghai General Hospital, Shanghai Jiao Tong University School of Medicine, Shanghai, China.; 2 Shanghai Key Laboratory of Pancreatic Diseases, Shanghai Jiao Tong University School of Medicine, Shanghai, China.; 3 Department of Radiology, Shanghai General Hospital, Shanghai Jiao Tong University School of Medicine, Shanghai, China.

**Keywords:** YBX1, fatty acid translocase CD36, lipid accumulation, hepatic steatosis, MASLD

## Abstract

Metabolic dysfunction-associated steatotic liver disease (MASLD) is a common chronic liver disorder mainly caused by an imbalance in lipid homeostasis. Y-box binding protein 1 (YBX1) participates in multiple pathophysiological processes, including embryonic development, tissue repair, liver disorders, and energy metabolism. The objective of this study is to investigate the mechanisms underlying MASLD and characterize the role of YBX1 in MASLD. A positive correlation between hepatic YBX1 expression and MASLD using single-cell sequencing data and human liver samples was observed. Hepatocyte-specific YBX1 deficiency ameliorates MASLD in a mouse model generated by subjecting *YBX1*-KO^hep^ and LOXP mice to a high-fat-cholesterol and high-fructose diet. Subsequently, the role of YBX1 in the hepatic lipid deposit was assessed by using primary hepatocytes and by performing transmission electron microscopy and biological and histological analyses. Mechanistically, the elevated YBX1 expression enhances the CD36 expression and its membrane localization by directly binding to the promoter of CD36. Furthermore, CD36 promotes the expression of YBX1 under lipid stimulation. The YBX1/CD36 positive feedback loop facilitates hepatic lipid accumulation. The up-regulation of CD36 attenuated the reduction of hepatic steatosis mediated by hepatic YBX1 deficiency in MASLD mouse models. These findings suggest that YBX1 is essential for hepatic lipid homeostasis. This study reveals a novel mechanism of liver steatosis and shows that targeting YBX1 may represent a potential approach for MASLD treatment.

## Introduction

Metabolic dysfunction-associated steatotic liver disease (MASLD), formerly known as nonalcoholic liver fatty disease (NAFLD), is the most prevalent chronic liver disease worldwide 1,2, affecting 38% of adults and 13% of children and adolescents worldwide 2,3. MASLD encompasses a wide spectrum of metabolic liver diseases, ranging from isolated liver steatosis to metabolic dysfunction-associated steatohepatitis, fibrosis, and cirrhosis 4. Hepatic steatosis, an early and critical clinical manifestation in the progression of MASLD, is associated with an elevated risk of systemic metabolic disorders, including type 2 diabetes mellitus (T2DM), hypertension, and dyslipidemia 5. Recently, a phase 3 trial reported that resmetirom, a thyroid hormone receptor beta (THR-β)-selective agonist, improved nonalcoholic steatohepatitis (NASH) or fibrosis resolution in NASH patients with liver fibrosis 6,7. However, NASH resolution or hepatic fibrosis could be improved in only fewer than 30% of patients treated with resmetirom, implying that there are therapeutic targets other than THR-β that need to be identified. Therefore, more efforts are needed to identify novel target molecules involved in the pathophysiology of MASLD.

Hepatocytes are crucial for sustaining hepatic and systemic lipid homeostasis under normal physiological conditions 8. The hallmark pathogenic feature of MASLD is the accumulation of lipids in hepatocytes 9. The potential sources of hepatic lipids include an increased influx of plasma-derived non-esterified fatty acids (NEFAs) into hepatocytes, elevated de novo lipogenesis (DNL), and the spillover of dietary fatty acids into the plasma following absorption from the intestine 9. Lipid accumulation in hepatocytes can be regulated by a pool of regulators, including hormones, cytokines, and transcription factors 10. A series of transcription factors, including upstream stimulatory factors (USFs), sterol regulatory element-binding protein 1C (SREBP1C), liver X receptors (LXRs), and carbohydrate-responsive element-binding protein (ChREBP), play crucial roles in the accumulation of hepatic lipid 11. However, many aspects of liver steatosis still remain poorly understood. Further research is needed to elucidate the mechanisms underlying MASLD.

Y-box binding protein-1 (YBX1) is a pleiotropic protein that can directly bind to RNA/DNA to modulate gene expression 12. It plays vital roles in various biological processes and pathological conditions, including embryonic development, cell proliferation, tissue repair, and tumor progression 12-15. As a DNA-binding protein, YBX1 could regulate the expression of FOXA1 and estrogen receptor α at the transcriptional level, thereby promoting the progression of breast, lung, and prostate cancers 16. YBX1 also functions as a “reader” of the m5C modification signal and maintains the mRNA stability of ORAI2, which contributes to the peritoneal metastasis of gastric cancer in high-fat microenvironments 17. In a previous study, we demonstrated that YBX1 regulates the proliferation of hepatic progenitor cells and aggravates liver fibrogenesis 18-20. In addition, it is involved in the exacerbation of liver fibrosis by increasing chromatin accessibility 21. It plays a crucial role in the regulation of metabolic processes in adipose tissue. Notably, it is involved in maintaining insulin sensitivity in adipocytes by binding itself to the phosphatase protector Alpha4 and regulating the expression of the protein Tyr phosphatase 1B, which in turn controls Tyr phosphorylation of the insulin receptor 22. Rabiee *et al.* identified YBX1 as a physiologically induced key factor that facilitates the formation of beige adipocytes in mice, thereby contributing to adaptive thermogenesis 23. However, the role of YBX1 in MASLD remains unknown.

This study aims to demonstrate that YBX1 is highly expressed in MASLD liver tissue and exerts a significant impact on lipid accumulation. Bioinformatics approaches were utilized to explore the relationship between YBX1 expression and hepatic steatosis, and to identify potential downstream genes. A conditional gene knockout mouse was generated to demonstrate the role of YBX1 in the pathogenesis of MASLD. The study results enable us to further understand the mechanism through which YBX1 regulates the progression of MASLD and demonstrate that YBX1 could be a potential candidate therapeutic target for MASLD treatment.

## Materials and methods

### Human liver samples

Liver biopsy samples were collected from patients with MASLD (*n* = 20). Normal liver tissues were obtained from the non-pathological regions of the liver of patients with hepatic hemangioma who underwent hepatectomy (*n* = 10). All procedures involving human samples were approved by the Ethics Committee of Shanghai General Hospital, following the 1975 Declaration of Helsinki guidelines. Written informed consent was obtained from each participant.

### Animal experiments

Eight-week-old C57BL/6J male mice were purchased from the Cyagen Company. All mice were maintained in a controlled facility (23±3°C, 30-70% humidity, 12 h light/dark cycle) and were provided with sterile food and water. The experiments were approved by the Animal Care and Use Committee of Shanghai General Hospital. The normal chow diet (Chow, 14.9% of calories from fat, 62.4% from carbohydrate, 22.7% from protein) was purchased from Shanghai Xietong (Cat: 1010082, Shanghai, China). The MASLD model was developed by administering a high-fat-cholesterol and high-fructose diet (HFCFD; 40% of calories from fat, 2% from cholesterol, and 20% from fructose; Research Diet D09100310).

Hepatocyte-specific *YBX1* knockout mice (*YBX1*-KO^hep^) were generated by crossing *YBX1*^flox/flox^ (LOXP) mice with an estrogen receptor-tagged albumin promoter-driven Cre (Alb-Cre^ERT2^) recombinase transgenic mouse in a C57BL/6 background. Genetype was validated through the polymerase chain reaction (PCR) by using the following primers: forward: 5'-GAAGCAGAAGCTTAGGAAGATGG-3'; reverse: 5'-TTGGCCCCTTACCATAACTG-3'. To induce recombination, 7-week-old mice were intraperitoneally injected with tamoxifen (Sigma, 10540-29-1) dissolved in peanut oil (Sigma, 8001-30-7) at a dosage of 40 mg/kg per day for five consecutive days.

*YBX1*-KO^hep^ and LOXP mice (*n* = 6 per group) were fed with Chow or HFCFD for 18 weeks. For the adeno-associated virus-mediated overexpression of CD36, *YBX1*-KO^hep^ (*n* = 12) and their littermates (*n* = 12) were administered either AAV8-vector or AAV8-CD36 (200 μL of saline containing 1×10^12^ viral genomes) via tail vein injection at week 14 of the modeling diet. They were then euthanized 28 days after injection. Murine liver tissues and serum were collected and stored at -80 °C for further examination.

### Isolation of primary hepatocytes, macrophages and Dendritic cells

The primary hepatocytes were isolated following the protocol described previously 24. Briefly, a perfusion buffer was prepared by mixing 50 mL of Hanks' balanced salt solution (HBSS, without Ca^2+^/Mg^2+^, Gibco, 1417509), 500 µL of 7.5% NaHCO_3_, and 71.17 µL of 0.5 M ethylenediaminetetraacetic acid (EDTA, Thermo Fisher, AM9260G). The digestion buffer was formulated with 30 mL of HBSS containing Ca^2+^/Mg^2+^ (Gibco, 14025134), 300 µL of 7.5% NaHCO_3_, 60 µL of 2.5 M CaCl_2_, and collagenase type 2 (Worthington, LS004176). After the mice were anesthetized, their abdominal and thoracic cavities were exposed, and a syringe needle was inserted into the right atrium and the portal vein was incised. Perfusion buffer was injected at 1-2 mL/min for 5 min, followed by the infusion of the digestive buffer into the liver at 1 mL/min for approximately 5 min until the tissue softened. Next, the hepatic tissue was transferred into a 10-cm dish, and the digestion buffer was added subsequently. The mixture was incubated at 37 °C for 3 min, followed by filtration into a 50-mL centrifuge tube. The mixture was centrifuged at 50*g* for 3 min, and the pellet was resuspended in DMEM. The centrifugation and resuspension procedures were performed twice, and the cells became ready for the subsequent experiments. Hepatic monocytes were isolated from the mixture by density gradient centrifugation after removal of the pellet. Subsequently, liver macrophages and dendritic cells (DCs) were purified by cell sorting using the mouse F4/80 positive selection kit (Thermo, 8802-6863-74) and the CD11c positive selection kit (Thermo, 8802-6861-74), respectively.

### Cell culture and transfections

The immortalized mouse liver cell line AML12 and the HEK293T cell line were purchased from Wuhan Pricella Biotechnology. AML12 cells were cultured in the DMEM/F12 medium (Gibco, A4192001) supplemented with 10% fetal bovine serum (FBS) (Gibco, A5669701), 40 ng/mL of dexamethasone (Sigma, D-085), and 0.45% insulin-transferrin-sodium selenite media supplement (Sigma, I3146). The resulting AML12 cells were then treated with palmitic acid (PA; MCE, 57-10-3) to establish a model of cellular steatosis. HEK293T cells were maintained in DMEM (Gibco, 11965092) with 10% FBS. Primary hepatocytes were cultured in the hepatocyte medium (Shanghai ZhongQiaoXinZhou Biotechnology, 5201). All cell lines were incubated at 37 °C in a humidified atmosphere containing 5% CO_2_.

The transfection of pCDH plasmids expressing YBX1 and CD36 was performed using Lipofectamine 3000 reagent (Invitrogen, L3000008). For stable knockdown, short-hairpin RNA (shRNA) plasmids targeting YBX1 were purchased from Shanghai Genechem. The shRNA sequences targeting YBX1 and the negative control were as follows: sh-*YBX1* (5'-AGAGCAAGGTAGACCAGTGA-3'); negative control (5'-TTCTCCGAACGTGTCACGT-3'). Lentivirus was produced using 293T packaging cells and transfected into target cell lines in the presence of 6 µg/mL polybrene for 24 h. The transfected cells, along with their control counterparts, were selected using 2 µg/mL of puromycin for 2 weeks. For conducting AKT signaling pathway validation experiments, the cells were treated with a CD36 inhibitor (sulfosuccinimidyl oleate sodium, E2988, 50 μM), an AKT agonist (SC79, S7863, 8 μg/mL), and an AKT inhibitor (MK2206, S1078, 3 μM) (Selleck Company). The knockdown efficiency of YBX1 was assessed by qRT-PCR and Western blotting.

### Statistics and reproducibility

Statistical analysis was conducted using GraphPad Prism 9.0 (GraphPad Software), with data presented as mean ± SD. Statistical significance was defined as *P < 0.05, **P < 0.01, ***P < 0.001 and ****P < 0.0001. Normality was assessed using the Kolmogorov-Smirnov test, Anderson-Darling test, D'Agostino-Pearson omnibus test, or Shapiro-Wilk test. Homogeneity of variances was tested using an F-test (*P* > 0.05). An unpaired two-tailed Student's *t*-test was used for comparisons between two groups of normally distributed data. Welch's correction was applied for groups with unequal variances. One-way ANOVA followed by Tukey's test was used for multiple group comparisons. The correlation between YBX1, CD36, and lipid droplet was analyzed using linear correlation and regression.

## Results

### YBX1 positively correlates with MASLD

**Figure 1:**
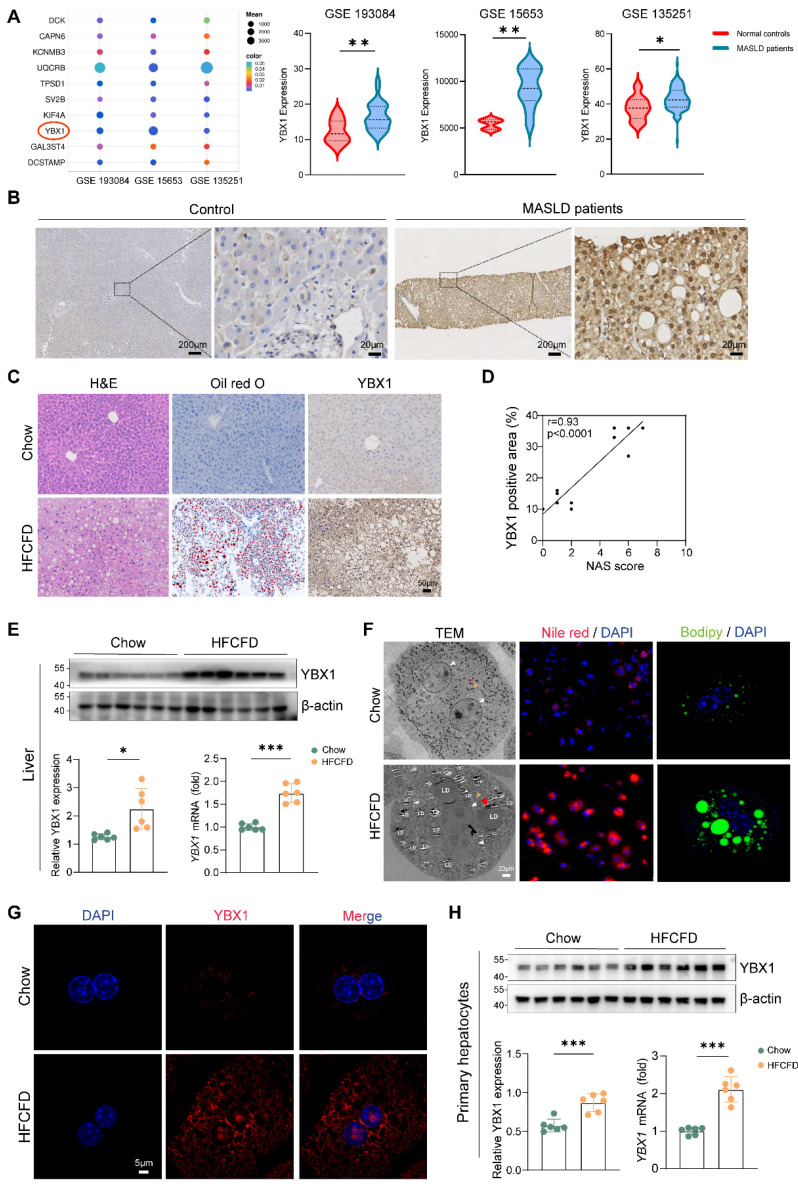
** Upregulation of YBX1 in mice and patients with MASLD.** A. Bubble plot representation of differentially expressed genes between MASLD patients and controls shared by GEO Datasets GSE 193084, GSE15653 and GSE 135251(left panel). Expression of YBX1 in each dataset was shown in the right panel. B. Representative images of IHC staining of YBX1 in liver sections from MASLD patients (n=20) and controls (n=10). Scale bar, 200μm or 20μm as indicated. C. Representative images of H&E, Oil Red O and YBX1 IHC staining of liver sections from mice fed with Chow or HFCFD (Scale bar, 50μm), n=6 per group. D. Correlation analysis of YBX1 IHC score and NAS score from mice fed with Chow or HFCFD, n=6 per group. E. Western blotting analysis of YBX1 expression (top panel) and RT-qPCR analysis of YBX1 expression (bottom right panel) of liver tissues obtained from mice fed with Chow or HFCFD. The statistical analysis of Western blotting is shown on the bottom left panel n = 6 per group. F. Representative images of TEM, Nile red (red) staining and Bodipy (green) staining of primary hepatocytes isolated from mice fed with Chow or HFCFD (Scale bar, 20μm). White triangle refers to the nucleus, red triangle refers to lipid droplets and yellow triangle refers to mitochondria. Scale bar, 30μm or 5μm as indicated. G. Representative immunofluorescence images of YBX1(red), and DAPI (blue) stained primary hepatocytes isolated from mice fed with Chow or HFCFD (Scale bar, 5μm). n=6 per group. H. Western blotting analysis of YBX1 expression (top panel) and RT-qPCR analysis of YBX1 expression (bottom right panel) of primary hepatocytes isolated from mice fed with Chow or HFCFD. The statistical analysis of Western blotting is shown on the bottom left panel. n=6 per group. LDs, lipid droplets. Data are presented as mean ± SD, with biologically individual data points shown. *p < 0.05, **p < 0.01, ***p < 0.001, ****p < 0.0001. P values were determined by linear correlation and regression analyses (D), unpaired two-tailed Student's t-test with Welch's correction (E and right panel of H) or unpaired two-tailed Student's t-test (right panel of A, left panel of H). HFCFD, high-fat-cholesterol and high-fructose diet; LDs, lipid droplets; TEM, Transmission electron microscopy.

To investigate whether YBX1 is involved in MASLD, we first analyzed the gene expression profile of patients with MASLD through GEO datasets (GSE193084, GSE15653, and GSE135251) and found that YBX1 is among the higher expressed candidates (Figure 1A). To demonstrate the clinical significance of YBX1, we compared its expression levels in both MASLD and control groups. IHC staining results showed that YBX1 was higher in both the cytoplasm and nucleus of hepatocytes in patients with MASLD than that of the control group (Figure 1B and Supplemental Figure 1A, S1A). Notably, correlation analysis showed that the IHC scores of YBX1 were positively correlated with the NAS scores (Figure S1B).

To recapitulate the key features of human MASLD, the C57BL/6J mice were subjected to HFCFD. H&E and oil red O staining revealed much higher lipid accumulation in MASLD mice (HFCFD group) than that of the controls (chow group) (Figure 1C). In agreement with the above results, compared to the chow group, the expression of YBX1 increased at both the mRNA and protein levels in the HFCFD group (Figure 1C-E, S1C).

Western blotting experiments and scRNA-seq (GSE129516) analysis showed that the YBX1 expression was predominantly high in the hepatocytes of the HFCFD-fed mice (S1D-F). In addition, the primary hepatocytes were isolated to focus on the effects of YBX1 on hepatic lipid homeostasis. The primary hepatocytes of the HFCFD group showed a significant up-regulation of YBX1 protein expression in both the cytoplasm and the nucleus, which was associated with increased lipid droplet accumulation, as visualized by PCM, TEM, Nile Red, and Bodipy staining (Figure 1F-G, S1G, H). Increased expression of YBX1 in primary hepatocytes from MASLD mice was further confirmed by Western blotting and qRT-PCR (Figure 1H). Likewise, the YBX1 levels exhibited a positive correlation with the lipid droplet content, as assessed by Bodipy staining in hepatocytes (Figure S1I). Collectively, these results demonstrated that hepatic YBX1 expression was positively correlated with MASLD.

### Hepatocyte-specific *YBX1* deficiency ameliorates hepatic steatosis

**Figure 2:**
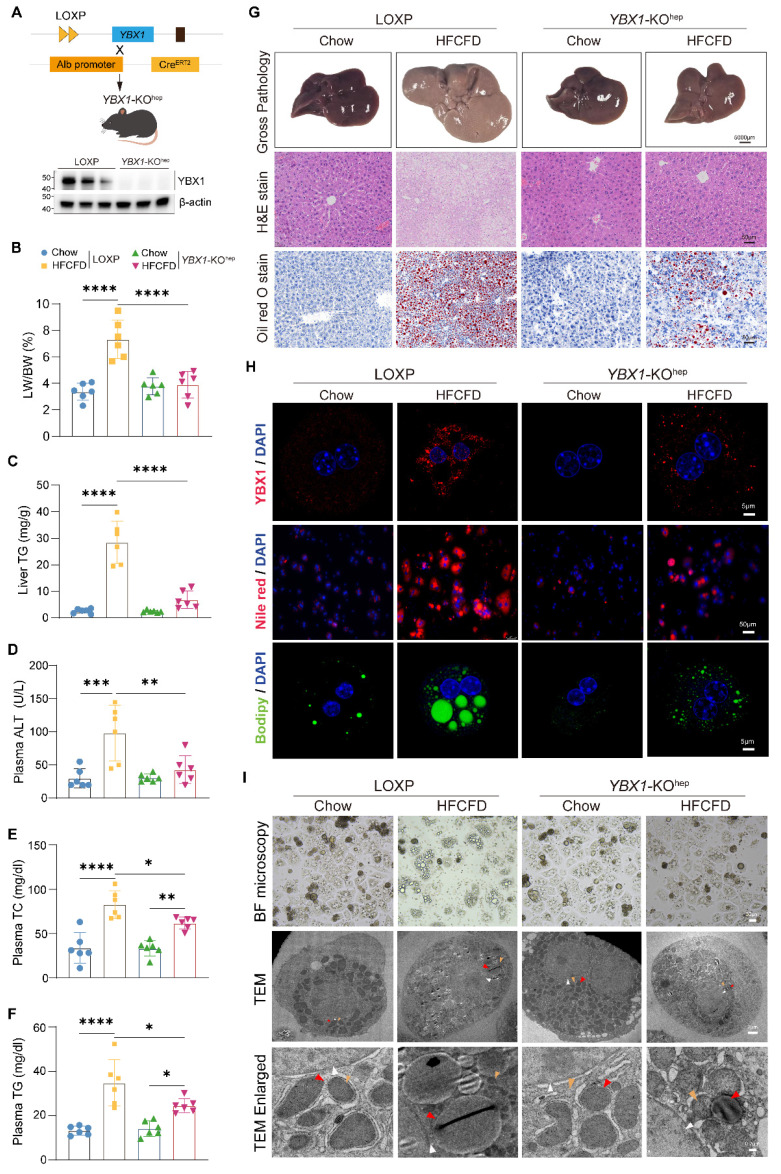
** Hepatocyte-specific YBX1 deletion ameliorates hepatic steatosis.** A. Schematic illustration of generating hepatocyte-specific YBX1 knockout mice (*YBX1*-KO^hep^) (top panel). Western blotting analysis of YBX1 expression in the primary hepatocytes isolated from LOXP and *YBX1*-KO^hep^ mice (bottom panel), n=3 mice per group. B. LW/BW in LOXP and *YBX1*-KO^hep^ fed with Chow or HFCFD, n=6 mice per group. C. Liver levels of TG in LOXP and *YBX1*-KO^hep^ mice fed with Chow or HFCFD, n=6 mice per group. D. Plasma levels of ALT in LOXP and *YBX1*-KO^hep^ mice fed with Chow or HFCFD, n=6 mice per group. E. Plasma levels of TC in LOXP and *YBX1*-KO^hep^ mice fed with Chow or HFCFD. Liver TG in LOXP and *YBX1*-KO^hep^ mice fed with Chow or HFCFD, n=6 mice per group. F. Plasma levels of TG in LOXP and *YBX1*-KO^hep^ mice fed with Chow or HFCFD, n=6 mice per group. G. Representative Gross pathology (scale bar, 5000μm), H&E and Oil Red O (scale bar, 50 μm) for liver tissues in LOXP and *YBX1*-KO^hep^ mice fed with Chow or HFCFD. H. Representative immunofluorescence images of YBX1 (red), DAPI (blue), Nile red and Bodipy staining of hepatocytes isolated from LOXP and *YBX1*-KO^hep^ mice fed with Chow or HFCFD (scale bar, 5μm or 50μm as indicated). I. Bright-field microscope (top panel, scale bar, 30μm) and TEM (middle and bottom panel) images of primary hepatocytes isolated from mice fed with Chow or HFCFD. White triangle refers to the nucleus, red triangle refers to lipid droplets, and yellow triangle refers to mitochondria. Scale bar, 30μm, 2μm or 0.2μm as indicated. Data are presented as mean ±SD, with biologically individual data points shown. *p < 0.05, **p < 0.01, ***p < 0.001, ****p < 0.0001. P values were determined by one-way ANOVA followed by Tukey's test (B-F). HFCFD, high-fat-cholesterol and high-fructose diet; TEM, Transmission electron microscopy; *YBX1*-KO^hep^, hepatocyte-specific *YBX1*-deficient mice.

YBX1 up-regulation in MASLD suggests that it might be involved in hepatic lipid accumulation. Thus, hepatocyte-specific *YBX1*-deficient mice (*YBX1*-KO^hep^) were generated by crossing LOXP with *Alb*-Cre^ERT2^ mice, as confirmed by qRT-PCR and Western blotting (Figure 2A and S2A). When fed with a normal chow diet, *YBX1*-KO^hep^ and LOXP mice did not show any significant differences in body weight; liver size; liver-to-body weight ratio (LW/BW); liver TG content; plasma ALT, AST, and lipid levels (including TG and TC); hepatic lipid accumulation; inflammation; and fibrosis (Figure 2B-G and S2B-F). However, in the case of HFCFD-induced MASLD model, the *YBX1*-KO^hep^ mice exhibited lower body weight, liver size, LW/BW, liver TG content, ALT, plasma lipid levels, and lesser accumulation of hepatic lipids than the LOXP mice (Figure 2B-G and S2B-F). Notably, compared with the LOXP mice, both the number and the size of lipid droplets reduced in the primary hepatocytes of the *YBX1*-KO^hep^ mice, as indicated by Nile Red, Bodipy staining, bright-field microscopy, and TEM analysis (Figure 2H and I). The quantity of lipid droplets decreased to one-seventh of the level observed in HFCFD-fed LOXP primary hepatocytes, and the mean diameter reduced from 2669.4 nm to 1158.6 nm (Figure S2G). Collectively, these data indicate that YBX1 deficiency reduces lipid accumulation in hepatocytes and mitigates liver steatosis in MASLD mice.

### YBX1 contributes to lipid accumulation and utilization in hepatocytes

**Figure 3:**
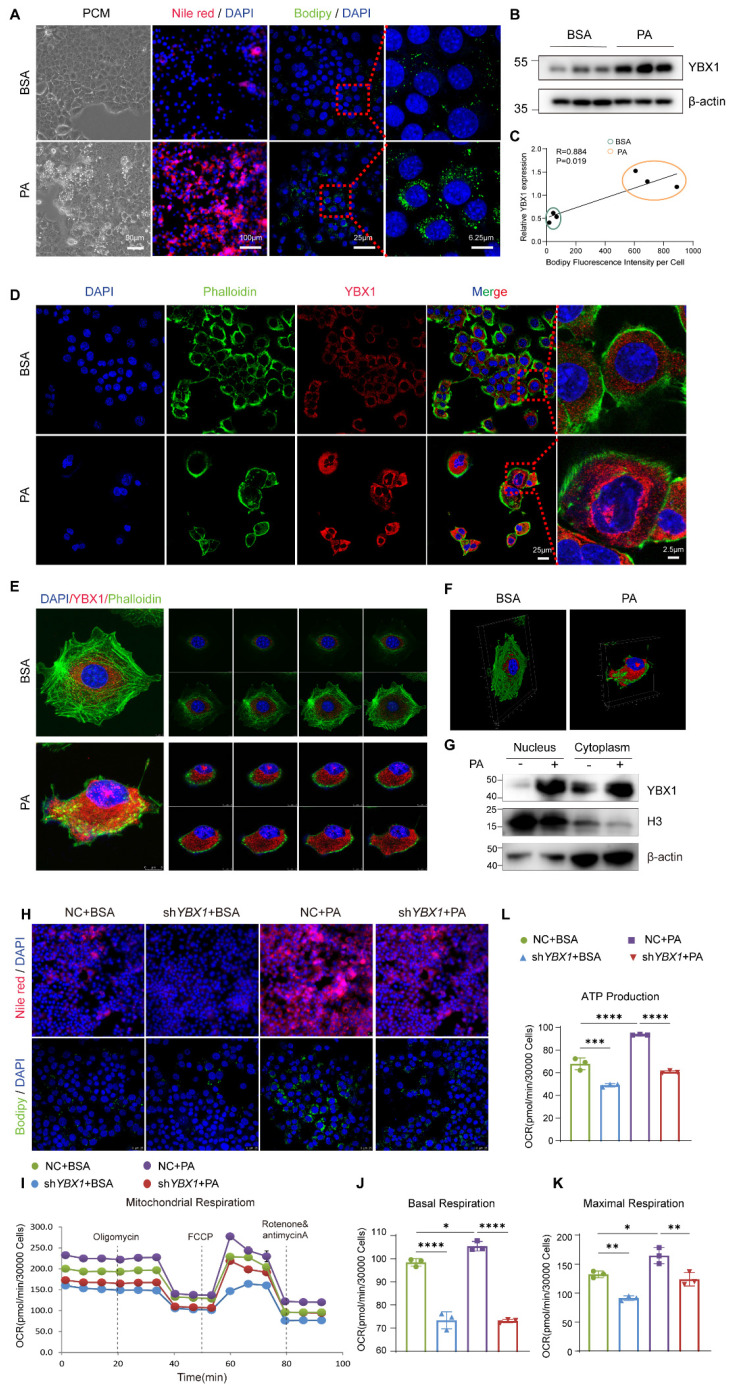
** YBX1 facilitates lipid accumulation and utilization in vitro.** A. Phase-contrast microscope (PCM), represented images of Nile red staining and Bodipy staining of AML12 cell line treated with 250μmol/L PA. Scale bar, 100μm, 90μm, 25μm or 6.25μm as indicated. B. Western blotting analysis of YBX1 expression in AML12 cells treated with PA, n = 3 biologically independent experiments. C. Correlation analysis of the expression level of YBX1 and Bodipy fluorescence intensity per cell, n = 3 biologically independent experiments. D. Representative immunofluorescence images of YBX1(red), and DAPI (blue), Phalloidin (green) of AML12 cells treated with PA (scale bar, 50μm or 2.5μm as indicated). E. Representative Z-stack fluorescence images of cell nuclei (blue), Phalloidin (green), and YBX1 (red) of AML12 cells treated with PA. The thickness of optical section is 5μm. F. The 3D reconstruction from segmented sections of Z-STACK. G. Nucleocytoplasmic separation and Western blotting analysis of YBX1 expression in AML12 cells treated with PA. Representative results from two independent biological experiments. H. Represented immunofluorescence images of Nile red staining and Bodipy staining of sh*YBX1* or NC cells treated with PA. I. Real-time Oxygen consumption rate (OCR) examination of sh*YBX1* or NC cells treated with PA. J. Basal OCR levels in sh*YBX1* or NC cells treated with PA. K. Maximal OCR levels in sh*YBX1* or NC cells treated with PA. L. ATP production levels in sh*YBX1* or NC cells treated with PA (I-L, n = 3 biologically independent experiments). Data are presented as mean ± s.d., with biologically individual data points shown. *p < 0.05, **p < 0.01, ***p < 0.001, ****p < 0.0001. P values were determined by linear correlation and regression analyses (C) and one-way ANOVA followed by Tukey's test (J-L). sh*YBX1* cells, cells with YBX1 stable knocked down by short hairpin RNA.

To gain insights into the role of YBX1 in lipid metabolism, we established an in vitro model by employing the PA-treated immortalized mouse hepatocyte cell line AML12. AML12 was first treated with 250 µM of PA to achieve higher effectiveness at a lower cost of lipid toxicity (Figure S3A-D). As expected, an accumulation of lipid droplets was observed in AML12 treated with 250 µM of PA, accompanied by an up-regulation of YBX1, and in genes associated with de novo lipogenesis, fatty acid transport, and lipolysis (Figure 3A, B, and S3E). The expression level of YBX1 was positively correlated with the area of lipid droplets per cell, as indicated by the intensity of Bodipy fluorescence (Figure 3C). Notably, the expression of YBX1 increased in both the cytoplasm and nucleus upon PA treatment (Figure 3D and G), which was consistent with the findings obtained using MASLD mice described above. Furthermore, Z-STACK imaging was applied to collect multiple images from a single cell at an optical section thickness of 5 μm. The results showed enhanced YBX1 expression in both the cytoplasm and the nucleus of each layer (Figure 3E).

The 3D reconstruction from the segmented sections of Z-STACK is shown in Figure 3F. We established a stable cell line of AML12 transfected with sh*YBX1* or control (Figure S3F). Upon PA treatment, a significant reduction in the number of intracellular lipid droplets was observed in the sh*YBX1* cells compared to that obtained using NC cells, as indicated by Nile Red and Bodipy staining (Figure 3H). Furthermore, when compared with that of NC cells, silencing YBX1 significantly reduced the levels of basal OCR, maximal OCR, and mitochondrial ATP synthesis upon PA treatment. Hence, it can be suggested that YBX1 is involved in the intake and utilization of long-chain-fatty acids in hepatocytes (Figure 3I-L). These findings suggest that the expression of YBX1 is up-regulated in response to fatty acid treatment, and that YBX1 might contribute to lipid accumulation, intake, and utilization in AML12 cells.

### YBX1 facilitates lipid accumulation in hepatocytes by up-regulating CD36

**Figure 4:**
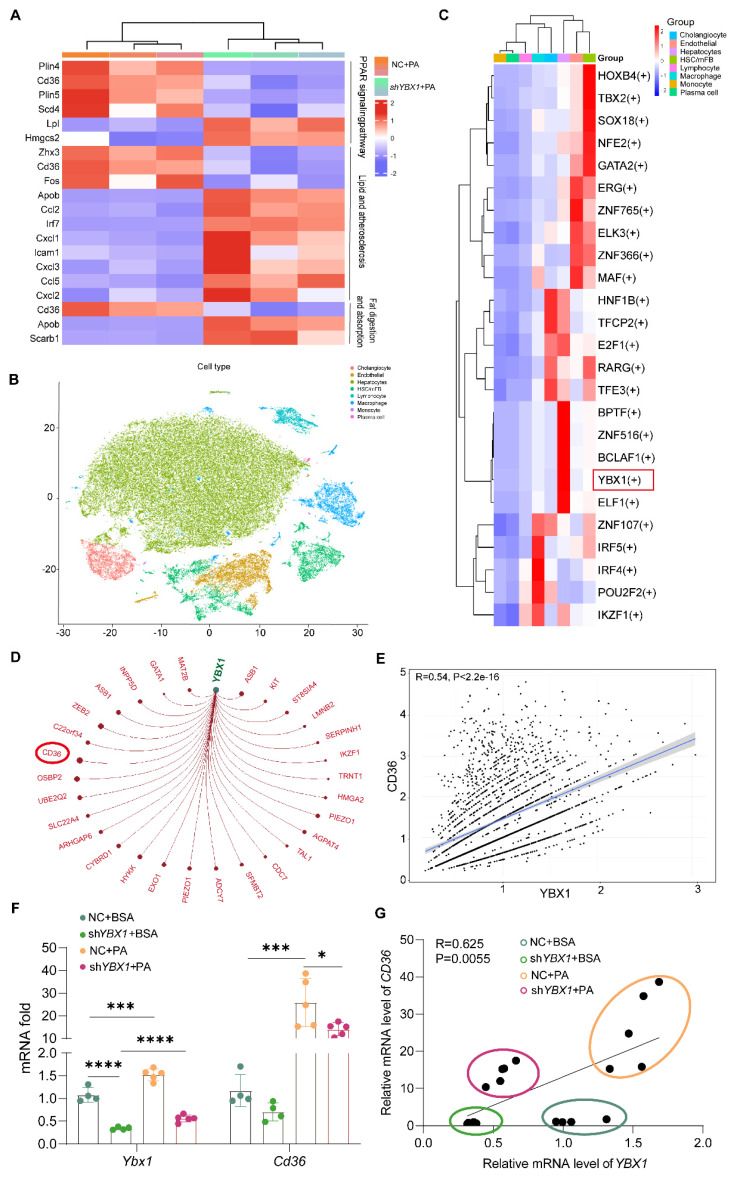
** YBX1 is involved in *CD36-*mediated lipid accumulation in hepatocytes.** A. Enriched KEGG pathway analysis of lipid metabolism pathway comparing the transcriptomic profiles of NC and sh*YBX1* cells. B. t-SNE plot showing the clustering of individual cells (n=27,914) based on the single-cell transcriptomic profile of GSE212837 dataset. Cells are colored according to their identified cell types. C. Heatmap of transcription factor (TF) activities in different cell types in GSE212837 dataset predicted by Scenic. D. Network of targeted genes of YBX1 in GSE212837 dataset predicted by SCENIC. Node size indicates the strength of the interaction between YBX1 and its targets. Only interactions with an adjusted p < 0.05 are shown. E. Correlation analysis of *CD36* expression and *YBX1* expression in GSE212837 dataset. F. The mRNA expression level of* YBX1* and *CD36* in sh*YBX1* or NC cells treated with PA (n = 4, NC + BSA; n = 4, sh*YBX1* + BSA; n = 5, NC + PA; n = 5, sh*YBX1* + PA). G. Correlation analysis of *YBX1* and *CD36* expression in sh*YBX1* or NC cells treated with PA. Data are presented as mean ± SD, with biologically individual data points shown. *p < 0.05, **p < 0.01, ***p < 0.001, ****p < 0.0001. P values were determined by one-way ANOVA followed by Tukey's test (F) and linear correlation and regression analyses (G). sh*YBX1* cells, cells with YBX1 stable knocked down by short hairpin RNA.

To clarify the molecular mechanisms through which YBX1 contributes to lipid metabolism, we performed transcriptomic sequencing in both NC and sh*YBX1* cells. The down-regulation of YBX1 led to the induction of some specific genes and a reduced expression of other specific genes, including CD36. KEGG enrichment analysis showed that the lipid metabolism pathway, comprising the lipid and atherosclerosis pathway, PPAR signaling pathway, and fat digestion and absorption, was enriched (Figure 4A). Subsequently, SCENIC, a single-cell regulatory network inference and clustering tool, was applied to ascertain whether YBX1 functions as a transcription factor in hepatocytes and to identify its targeted genes. The analysis of the single-cell RNA sequencing data of liver tissues collected from patients with MAFLD (GSE212837) using SCENIC demonstrated that YBX1 may function as a transcription factor in hepatocytes (Figure 4B-C, S4A, and Table S1). Moreover, CD36 was predicted to be a target gene of YBX1 with the highest target score. The expression level of CD36 was positively correlated with that of YBX1 in the liver tissues of MASLD patients and in an in vitro steatosis model (Figure 4D-G and Table S2).

**Figure 5:**
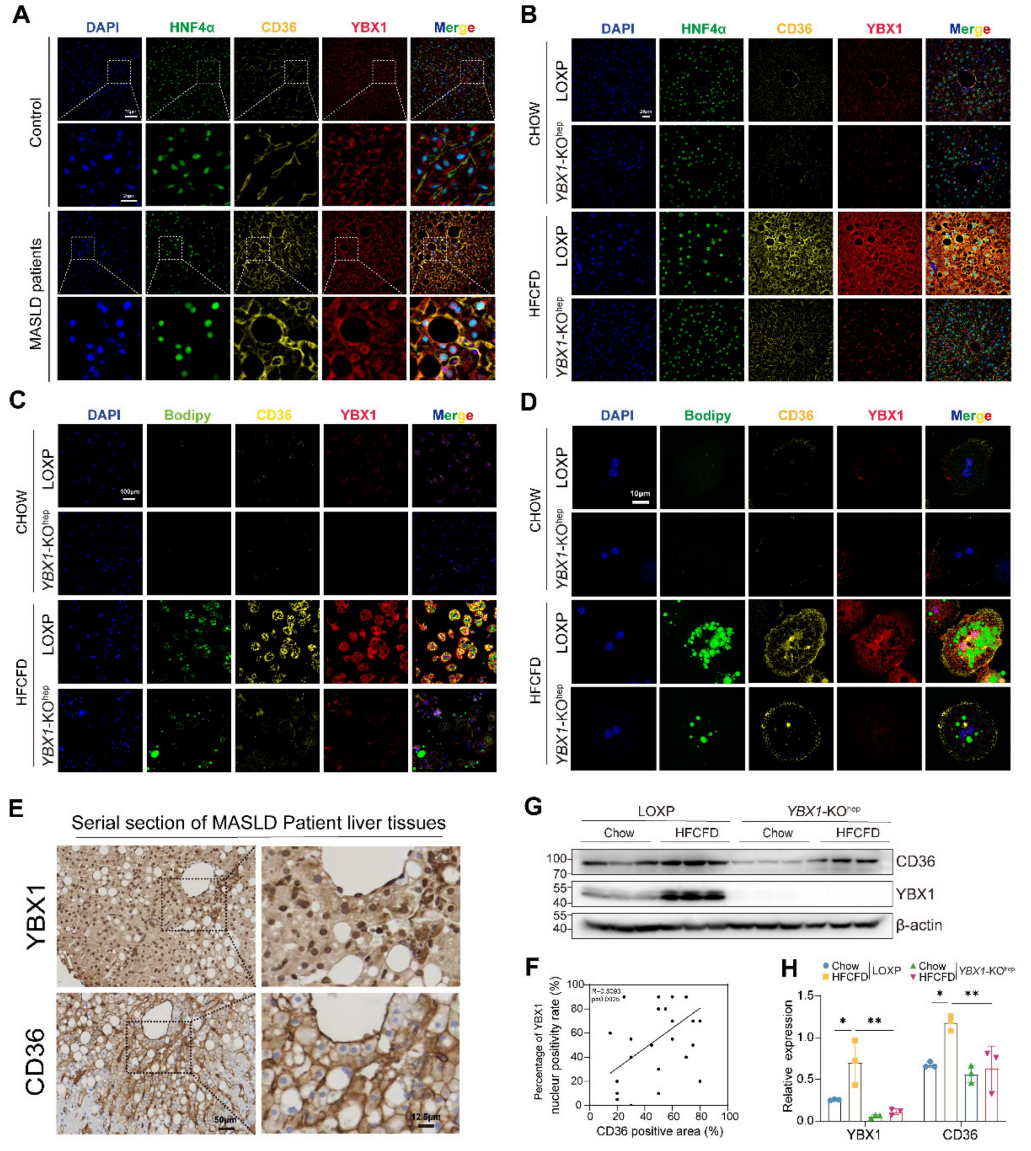
** Hepatic expression of CD36 increased along with enhanced nucleus YBX1 expression in MASLD patients and mice.** A. The presented multiparametric fluorescence imaging of cell nuclei (blue), HNF4α(green), CD36 (yellow) and YBX1 (red) in liver tissues of MASLD patients. White triangle indicates YBX1 expression in the nucleus of hepatocyte. Scale bar, 75μm or 25μm as indicated. B. Representative multiparametric fluorescence imaging of cell nuclei (blue), HNF4α(green), CD36 (yellow) and YBX1 (red) in liver tissues of LOXP and *YBX1*-KO^hep^ mice fed with Chow or HFCFD. White triangle indicates YBX1 expression in the nucleus of hepatocyte. Scale bar, 10μm or 25μm as indicated. C-D. Representative multiparametric fluorescence imaging of cell nuclei (blue), CD36 (yellow) and YBX1 (red) in primary hepatocytes isolated from LOXP and *YBX1*-KO^hep^ mice fed with Chow or HFCFD (C). Enlarged images for a single hepatocyte from (C) were shown in (D). Scale bar, 10μm or 100μm as indicated. E. Representative IHC staining of YBX1 and CD36 in serial sections of liver tissues from MASLD patients. Scale bar, 12.5μm or 50μm as indicated. F. Correlation analysis of CD36 IHC score and percentage of YBX1 nuclear positivity rate (n = 11 patients). G. Western blotting analysis of YBX1 and CD36 expression in liver tissue of LOXP and *YBX1*-KO^hep^ mice fed with Chow or HFCFD. H. The statistical analysis of (G). (G, H n = 3 mice.). Data are presented as mean ± SD, with biologically individual data points shown. *p < 0.05, **p < 0.01, ***p < 0.001. P values were determined by linear correlation and regression analyses (F) and one-way ANOVA followed by Tukey's test (H). HFCFD, high-fat-cholesterol and high-fructose diet; *YBX1*-KO^hep^, hepatocyte-specific *YBX1*-deficient mice.

We further validated that hepatic CD36 expression increased along with enhanced YBX1 expression in MASLD patients and mice (Figure 5A, B). Moreover, the YBX1 expression was observed in the nucleus of hepatocytes adjacent to adipose vacuoles in MASLD patients and mice but not in the control group, implying that high fat stimulation may facilitate the entry of YBX1 into the nucleus where it would function as a transcription factor (Figure 5A, B). Specifically, compared with LOXP mice, the targeted ablation of YBX1 in hepatocytes resulted in a significant reduction in lipid droplet vacuolation, accompanied by a down-regulation of CD36 expression (Figure 5B). In the primary hepatocytes isolated from *YBX1*-KO^hep^ mice, the expression of CD36 was also down-regulated, along with reduced YBX1 expression (Figure 5C-D and G-H). Furthermore, by examining the serial sections of the liver tissues of MASLD patients, we found that the expression of YBX1 in the nucleus of hepatocytes was positively correlated with that of CD36 (Figure 5E-F). These results suggest that YBX1, as a transcription factor, may play a role in CD36-mediated lipid accumulation in hepatocytes.

### YBX1 regulates CD36 through a positive feedback loop

**Figure 6:**
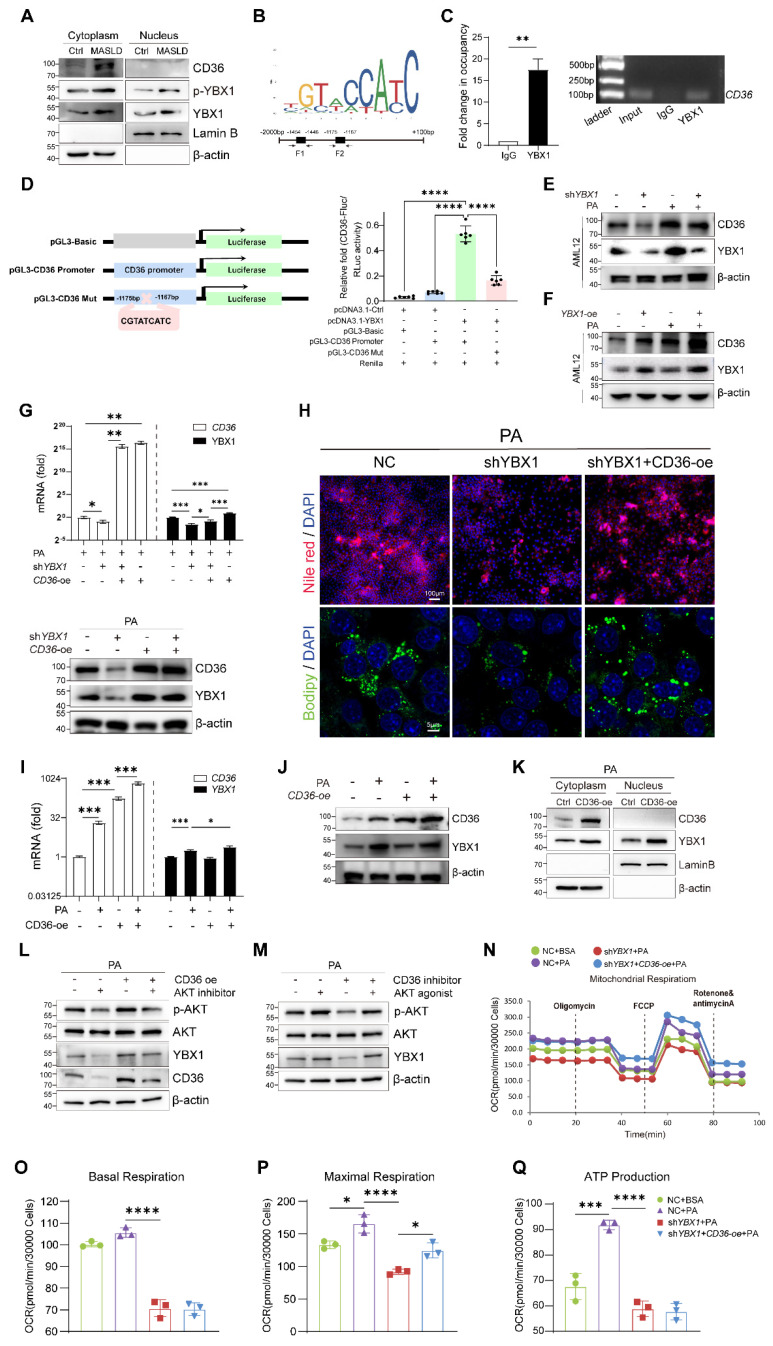
** YBX1 regulates CD36 through a positive feedback loop.** A. Nucleocytoplasmic separation and Western blotting analysis of YBX1, phosphorylated YBX1 and CD36 expression in primary hepatocytes isolated from mice fed with Chow or HFCFD. Representative results from two independent biological experiments. B. Binding Motif and two putative binding fragments of YBX1 in the promoter of CD36 predicted by JASPAR. C. Binding of YBX1 to the promoter of CD36 identified by ChIP (n = 3 biologically independent experiments). The enrichment of YBX1 on the promoter of CD36 was examined by CHIP-qPCR assay (right panel). The statistical analysis was shown on the left panel. D. Schematic illustration of reconstructed plasmids (left panel). Luciferase activities in indicated groups (right panel). Representative result from two independent biological experiments with six technical replicates. E. Western blotting analysis of YBX1 and CD36 expression in sh*YBX1* or NC cells treated with or without PA. Representative results from two independent biological experiments. F. Western blot analysis of YBX1 and CD36 in AML12 cells overexpressing YBX1 in the presence or absence of PA-supplemented media. Representative results from two independent biological experiments. G. RT-qPCR and Western blotting analysis of YBX1 and CD36 expression level in sh*YBX1* or NC cells overexpressing CD36 with PA treatment (n = 3 biologically independent experiments). H. Represented images of Nile red and Bodipy staining of NC cells, sh*YBX1* cells and sh*YBX1* cells overexpressing CD36 under PA treatment. I. RT-qPCR analysis of *YBX1* and CD36 expression in AML12 cells overexpressing CD36 treated with or without PA (n = 3 biologically independent experiments). J. Western blotting analysis of YBX1 and CD36 expression level in AML12 cells overexpressing CD36 treated with or without PA. Representative results from two independent biological experiments. K. Nucleocytoplasmic separation and Western blotting analysis of YBX1 and CD36 expression in AML12 cells overexpressing CD36 treated with PA. Representative results from two independent biological experiments. L. Western blotting analysis of YBX1, CD36, AKT and phosphorylated AKT expression level in AML12 cells overexpressing CD36 treated with or without AKT inhibitor under PA stimulation. M. Western blotting analysis of YBX1, AKT and phosphorylated AKT expression level in AML12 cells treated with or without AKT agonist or CD36 inhibitor under PA stimulation. N. Real-time Oxygen consumption rate (OCR) examination of NC cells treated with or without PA and CD36-overexpressed sh*YBX1* cells treated with or without PA. O. Basal OCR levels in NC cells treated with or without PA and CD36-overexpressed sh*YBX1* cells treated with or without PA. P. Maximal OCR levels in NC cells treated with or without PA and CD36-overexpressed sh*YBX1* cells treated with or without PA. Q. ATP production levels in NC cells treated with or without PA and CD36-overexpressed sh*YBX1* cells treated with or without PA. (K-N, n = 3 biologically independent experiments). Data are presented as mean ± SD, with biologically individual data points shown. *p < 0.05, **p < 0.01, ***p < 0.001, ****p < 0.0001. P values were determined by one-way ANOVA followed by Tukey's test (D, G, I, O-Q). PGL3-CD36 Promoter, a luciferase reporter construct containing the CD36 gene promoter region; shYBX1 cells, cells with YBX1 stable knocked down by short hairpin RNA; YBX1-oe, pCDH plasmids overexpressing YBX1; CD36-oe, pCDH plasmids overexpressing CD36.

To clarify the molecular mechanisms through which the YBX1/CD36 axis contributes to lipid accumulation, nucleocytoplasmic separation results were obtained (Figure 6A), which verified that CD36 and YBX1 were markedly upregulated and the nuclear translocation of YBX1 was increased in MASLD. The phosphorylation status of YBX1 was assessed. The alteration of its phosphorylated form (pYBX1) was consistent with that of the total YBX1, indicating that the total YBX1 levels are sufficient to regulate this pathway and are not strongly correlated with the phosphorylated forms. To explore the role of YBX1 in regulating CD36, we initially predicted the binding site of YBX1 by searching the JASPAR database and identified two putative binding fragments of YBX1 (F1 and F2) in the promoter region of CD36 (Figure 6B). ChIP experiments were performed using AML12 cells to identify potential YBX1 response elements in the CD36 promoter region. ChIP-PCR confirmed the binding of YBX1 to a fragment (F1) in the CD36 promoter region. ChIP-qPCR was then performed, and the results confirmed a significant increase in the CD36 F1 promoter region (Figure 6C and S4B).

Dual-luciferase reporter assays were utilized to identify the specific YBX1 response element in the CD36 promoter region. F1 was deleted from the reconstructed plasmid pGL3-CD36 promoter to generate the mutant pGL3-CD36 Mut (Figure 6D). Next, pGL3-CD36 promoter or pGL3-CD36 Mut was co-transfected with pcDNA3.1-*YBX1* into HEK293T cells, followed by the examination of luciferase activity. As shown in Figure 6D, the pGL3-CD36 promoter construct exhibited a marked increase in luciferase activity compared to that shown by the control group. Conversely, the luciferase activity of the pGL3-CD36 Mut construct significantly diminished (*P* < 0.001), affirming the specificity of YBX1 binding to the CD36 promoter region. In AML12 cells, we reconfirmed that YBX1 regulates the transcription of CD36 (Figure S4C and Figure 6E and F). The experimental data demonstrated that both the mRNA and protein levels of CD36 were directly correlated with variations in YBX1 expression, exhibiting concomitant increases and decreases in expression.

To identify whether CD36 functions as a critical downstream effector of YBX1 in promoting lipid deposition, we transfected sh*YBX1* cells with CD36-overexpression plasmids and subsequently treated them with PA (Figure S4D and Figure 6G). The result showed that the CD36 overexpression partially restored the lipid droplet accumulation, which had been significantly diminished by YBX1 knockdown (Figure 6H).

An intriguing phenomenon is shown in Figure 6G: The overexpression of CD36 unexpectedly resulted in a significant increase in the YBX1 protein level under high-fat conditions. To determine whether this phenomenon is specific to the high-fat environment, we conducted experiments involving the overexpression of CD36 in both normal and PA-supplemented culture media for both NC and sh*YBX1* cells. The results showed that the CD36 overexpression boosts YBX1 expression upon PA treatment but not under normal conditions (Figure 6I-J). This result suggests that in the context of high-fat conditions, increased CD36 may enhance YBX1 expression via positive feedback, promoting lipid droplet accumulation. The nucleocytoplasmic separation results showed that CD36 overexpression boosts YBX1 expression and nucleus translocation upon sustained PA treatment (Figure 6K). We conducted more experiments to elucidate the molecular mechanism through which CD36 influences YBX1 expression. The results showed that CD36 enhances YBX1 expression through AKT activation, which facilitates the translocation of YBX1 into the nucleus, thereby promoting its own expression and establishing a positive feedback loop (Figure 6K-M). In addition, CD36 overexpression significantly reversed the decline of the maximum OCR but not that of basal OCR and mitochondrial ATP synthesis induced by YBX1 deficiency (Figure 6N-Q). These findings indicate that YBX1 binds to the promoter of CD36 and promotes its transcription, while CD36 enhances YBX1 expression via the activation of AKT under MASLD conditions. Hence, the YBX1/CD36 positive feedback loop facilitates hepatic lipid accumulation and MASLD.

### CD36 mediates YBX1-facilitated lipid accumulation and MASLD

**Figure 7:**
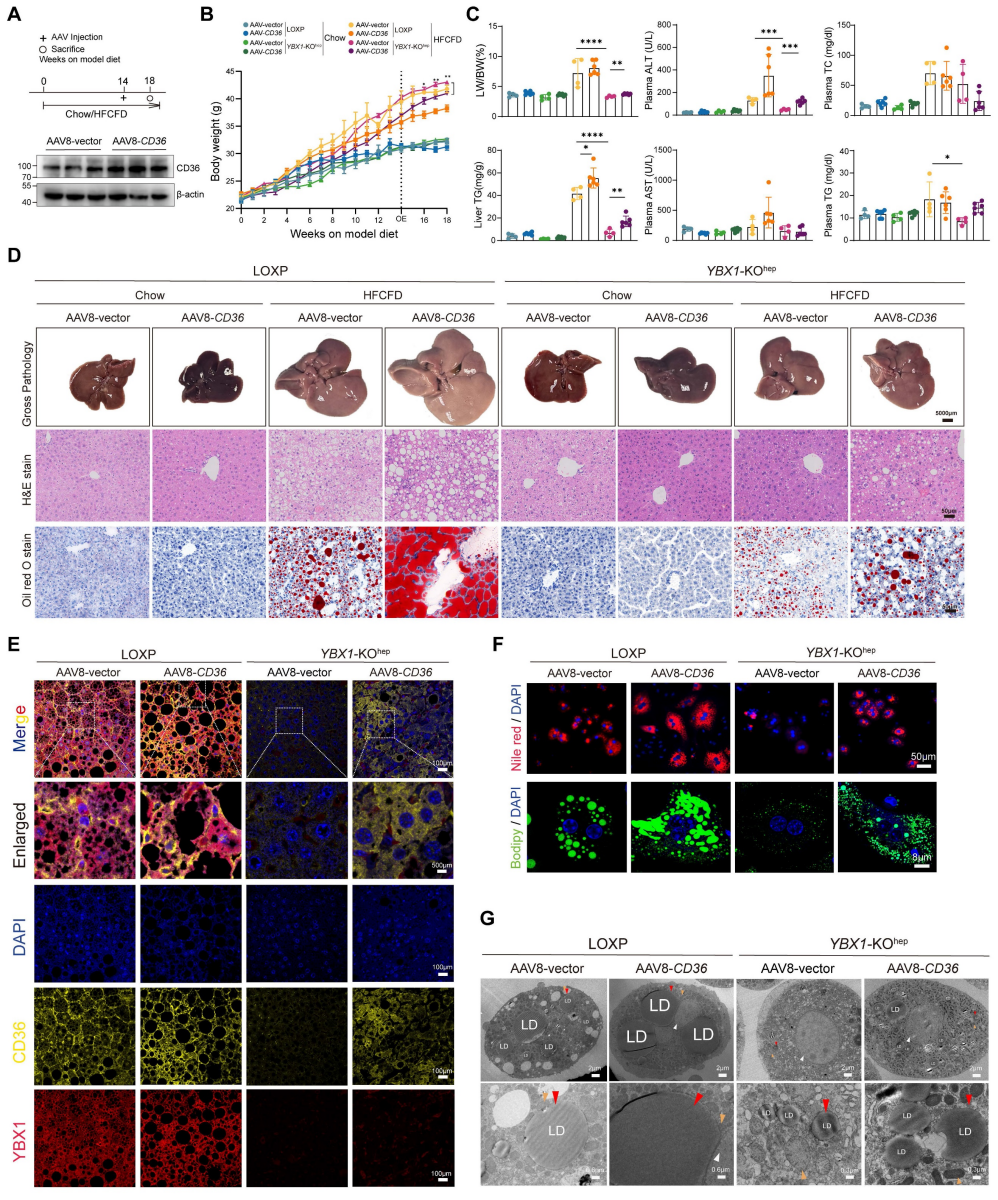
** CD36 mediates YBX1-facilitated lipid accumulation and MASLD.** A. Schematic illustration of experimental design for AAV8-*CD36* injection into LOXP and *YBX1*-KO^hep^ mice fed with Chow or HFCFD (top panel). After model diet feeding for 14 weeks, LOXP and *YBX1*-KO^hep^ mice were treated with AAV8-vector or AAV8-*CD36* via tail vein injection. The timeline of the experimental process was illustrated in the diagram. Western blotting analysis verified the efficiency of CD36 overexpression plasmid (bottom panel) (n=3 mice per group). B. Dynamics of body weight at indicated time points. n=4-6 mice per group. C. LW/BW, liver TG, plasma ALT, AST, TG and TC in LOXP and *YBX1*-KO^hep^ mice fed with Chow or HFCFD injected with AAV8-vector or AAV8-*CD36*. n=4-6 mice per group. D. Represented images of Gross pathology (scale bar, 5000μm), H&E and Oil Red O (scale bar, 50μm) for liver tissues of LOXP and *YBX1*-KO^hep^ mice fed with Chow or HFCFD injected with AAV8-vector or AAV8-*CD36*. E. Represented in multiparametric fluorescence imaging of YBX1 and CD36 expression in liver tissue from LOXP and *YBX1*-KO^hep^ mice fed with Chow or HFCFD injected with AAV8-vector or AAV8-*CD36*. F. Representative images of Nile red and Bodipy staining of primary hepatocytes isolated from LOXP and *YBX1*-KO^hep^ mice fed with Chow or HFCFD injected with AAV8-vector or AAV8-*CD36*. (Scale bar, 50μm or 8μm). G. Representative TEM images of primary hepatocytes isolated from mice LOXP and *YBX1*-KO^hep^ mice fed with Chow or HFCFD injected with AAV8-vector or AAV8-*CD36*. White triangle refers to the nucleus, red triangle refers to lipid droplets, and yellow triangle refers to mitochondria. (Scale bar, 2μm, 0.3μm, 0.6μm or 0.8μm). Data are presented as mean ± SD, with biologically individual data points shown. *p < 0.05, **p < 0.01, ***p < 0.001, ****p < 0.0001. P values were determined by unpaired two-tailed Student's t-test (B) and one-way ANOVA followed by Tukey's test (C). HFCFD, high-fat-cholesterol and high-fructose diet; *YBX1*-KO^hep^, hepatocyte-specific *YBX1*-deficient mice; AAV8-*CD36*, adeno-associated virus-mediated overexpression of CD36.

To further validate the regulatory effect of YBX1 on CD36 *in vivo*, we overexpressed CD36 by injecting AAV8-CD36 at the indicated times (Figure 7A and S4E, F). As expected, compared with the LOXP mice, a lower body weight, LW/BW, plasma ALT, and liver and plasma TG levels, as well as fewer lipid droplets in the liver, were observed in the HFCFD-fed *YBX1*-KO^hep^ mice (Figure 7B-D). Notably, CD36 overexpression negated the improvement of liver inflammation and lipid accumulation induced by *YBX1* knockout in the MASLD group. Subsequently, the reduction of lipid droplets observed in the YBX1 knockout group was reversed upon overexpression of CD36 (Figure 7E). Nile Red and Bodipy staining on primary hepatocytes demonstrated that the overexpression of CD36 abrogates the protective effects provided by the knockout of YBX1 under HFCFD conditions (Figure 7F), which was also confirmed by TEM (Figure 7G). These results show that CD36 mediates YBX1-facilitated lipid accumulation and MASLD.

## Discussion

MASLD is characterized by lipid accumulation in the liver and its subsequent inflammation and fibrogenesis 1. According to the traditional “two-hit” hypothesis, lipid accumulation is the first step in the development of MASLD 10. A number of transcription factors play a series of vital roles that result in the development of liver steatosis. YBX1 is associated with lipid metabolism in adipocytes 22,23. However, its specific function and clinical relevance in MASLD remain largely uncharacterized. This study showed that YBX1 is up-regulated in MASLD and positively correlated with the severity of hepatic steatosis. Further investigation indicated that the hepatocyte-specific deletion of YBX1 in HFCFD-fed mice ameliorates liver weight, hepatic lipid accumulation, liver biochemical parameters, serum lipid levels, and body weight. In addition, the study results revealed that YBX1 can transcriptionally modulate the expression of CD36 and enhance the plasma membrane localization. Moreover, lipid stimulation acts as a switch, promoting CD36 to enhance the expression of YBX1. Subsequently, the YBX1/CD36 positive feedback loop leads to lipid transport and accumulation in hepatocytes. Overexpression of CD36 partially restores hepatic lipid accumulation in *YBX1*-KO^hep^ mice fed with HFCFD. Hence, we can conclude that YBX1 enhances hepatic lipid uptake by positively regulating the transcription of CD36 and exerts a pivotal role in liver steatosis.

YBX1 is a highly conserved multi-functional protein and participates in a wide range of biological processes, including embryonic development, tissue repair, and tumor progression 12. With regard to liver disorders, the roles of YBX1 have been extensively explored in liver fibrosis and hepatocellular carcinoma. Previous studies have demonstrated that YBX1 is up-regulated in the fibrotic liver, and its knockdown ameliorates hepatic fibrosis in mouse models 25,26. Li *et al.* reported that the phosphorylation of YBX1 protein leads to the translation of proliferation-related genes, including cyclin D1 and cyclin E1, which subsequently enhances hepatocarcinogenesis 27. YBX1 was also reported as a critical factor in the regulation of metabolic processes in adipose tissue. Wu *et al.* found that YBX1 enhances thermogenesis through PINK1/PRKN-mediated autophagy in brown adipocytes 28.

A recent study demonstrated that YBX1 can enhance ULK1 mRNA stability and ULK2 transcription to promote ULK1/ULK2-mediated autophagy, resulting in the expansion of adipose tissue in mice 29. In this study, a higher expression of YBX1 was validated in MASLD patients based on the information obtained from the GEO datasets (GSE193084, GSE15653, and GSE135251). Consistently, we found that the YBX1 expression in hepatocytes increased in MASLD, both in mice and humans. Furthermore, our results indicated that the expression of YBX1 is positively correlated with the severity of hepatic steatosis. In addition, the hepatocyte-specific knockout of YBX1 reduced lipid accumulation and improved liver steatosis in the MASLD mouse model, implying that YBX1 is involved in the progression of MASLD. However, a recent study demonstrated that long non-coding RNA LNCHC regulates YBX1 stability to ameliorate MASLD progression in rats by stabilizing PNPLA3 mRNA 30. In contrast, our study initially validated the increased expression of YBX1 under fatty liver conditions using clinical samples and clinical databases. Subsequently, we employed hepatocyte-specific knockout mice to ascertain its function, as opposed to using rats without YBX1 gene knockout. For in vitro experiments, we utilized the immortalized mouse cell line (AML12) and mouse primary hepatocytes. In addition, we induced lipid deposition by treating cells with palmitic acid (PA). Previous studies used LO2 (human hepatocytes) and BRL-3A (rat hepatocytes) treated with a combination of oleic acid (OA) and PA. Therefore, it can be suggested that these disparities may have contributed to the inconsistency observed between the two studies.

YBX1 has been reported to play multiple roles as a transcription factor in physiological and pathological processes. In 1988, it was reported that YBX1 directly binds to an MHC II cis-acting element and negatively regulates the transcription of MHC II 31. In the present study, we found elevated expression levels of YBX1 in both the cytoplasm and the nucleus of hepatocytes in MASLD livers. These results indicate that YBX1 can function as a transcription factor in the context of hepatic steatosis. Previous studies have demonstrated that nuclear translocation of YBX1 occurs in both its total and phosphorylated forms 32,33. In this study, we evaluated the levels of both total YBX1 and its phosphorylated form (pYBX1) in the context of MASLD. Our results revealed that the changes in the phosphorylated form of YBX1 were consistent with those observed in the total YBX1, suggesting that YBX1 translocates to the nucleus through both its native and phosphorylated states. Nuclear localization and expression of YBX1 were further corroborated through Z-STACK imaging and nucleocytoplasmic separation followed by Western blotting analysis. The analysis of single-cell sequencing data (GSE212837) also indicated that YBX1 may function as a transcriptional regulator in hepatocytes. CD36 is identified as one of the top-ranked targeted genes.

CD36 is identified as a scavenger receptor for oxidized low-density lipoproteins in macrophages and as a membrane fatty acid transporter involved in the uptake of long-chain fatty acids 34,35. It is also expressed in adipocytes, cardiomyocytes, mononuclear phagocytes, and a subtype of epithelial cells. Increasing evidence suggests that CD36 is a key transporter of fatty acid influx from the circulating reservoir to hepatocytes, and is regarded as a critical participant in the onset and development of MASLD 36. CD36 is up-regulated in human livers with MASLD and its expression is positively correlated with the liver lipid content and disease progression 37,38. The present study confirmed that the expression of CD36 is relatively low in normal livers and high in steatotic livers in MASLD patients and mouse models. Consistent with the previous studies, our results also showed that CD36 was predominantly expressed at the plasma membrane of hepatocytes 38,39. Furthermore, our results indicated that the expression of CD36 on the plasma membrane is positively correlated with YBX1 expression in the hepatocyte nucleus. YBX1 can directly bind to the promoter of CD36. Hence, we can suggest that YBX1 can modulate hepatosteatosis by regulating the expression of CD36 at the transcription level. Interestingly, it was reported that YBX1 can bind to the coding sequence to promote CD36 mRNA decay and block the lipid uptake in macrophages, indicating that YBX1 plays a post-transcriptional regulatory role in CD36 expression and negatively modulates lipid uptake in macrophages 40. Therefore, in different cell types, YBX1 may be involved in the regulation of CD36 expression at different regulatory levels. In addition, the roles of YBX1 in lipid metabolism also vary due to cell type and stimulus. More experiments need to be performed to further reveal the regulatory roles of YBX1 in CD36 expression and lipid metabolism in multiple cell types, including adipocytes, macrophages, myocytes, and hepatocytes.

Notably, we found that the overexpression of CD36 could up-regulate YBX1 expression under PA stimulus. However, this phenomenon was not observed in the absence of PA. These findings indicate that lipid stimulation may act as a switch, initiating a positive feedback effect between lipid droplets, YBX1, and CD36. Increasing evidence suggests that besides being a fatty acid transporter, CD36 also functions as an important signal protein 34. After binding to the fatty acid, CD36 activates various signal effectors involved in lipid metabolism, including Src/LKB1/AMPK, ERK1/2, and VEGFR2/AKT. However, the molecular mechanism through which CD36 regulates the expression of YBX1 remains unclear. Previous studies have reported that AKT activation can facilitate the nuclear translocation of YBX1 41. Notably, the inhibition of AKT-mediated serine/threonine phosphorylation is associated with the reduced expression of YBX1 in adipocytes 22. Palmitic acid can promote the phosphorylation of AKT 42. Thus, there might be a mutual regulation of YBX1 and CD36 expression under excessive lipid-overload conditions. We propose that CD36 facilitates the transport of lipid droplets into hepatocytes, leading to the activation of AKT, which subsequently up-regulates the expression of YBX1. To validate this hypothesis, we conducted a series of experiments to elucidate the molecular mechanism underlying Ybx1 induction. Under MASLD conditions, the expression of CD36 is elevated because of the presence of excess lipids, which enhance the transport of lipids into the cytoplasm and activate AKT. The activation of AKT, in turn, increases YBX1 expression, which further upregulates CD36. Therefore, our findings suggest that YBX1 aggravates MASLD through a YBX1-CD36 positive feedback loop.

Our study is the first to verify that YBX1 positively modulates the transcription of CD36 and membrane localization of the protein, thereby promoting CD36-mediated fatty acid uptake. This result boosts the expression of YBX1, which leads to lipid accumulation in hepatocytes. Notably, targeting YBX1 in hepatocytes alleviates lipid accumulation and hepatic steatosis. These findings may provide insights into the understanding of hepatic lipid homeostasis and present a potential strategy to treat MASLD and related complications.

## Supplementary Material

Supplementary materials and methods, figures.

Supplementary table 1.

Supplementary table 2.

## Abbreviations

MASLD: Metabolic dysfunction-associated steatotic liver disease
NAFLD: Nonalcoholic liver fatty disease
YBX1: Y-box binding protein-1
HFCFD: high-fat-cholesterol and high-fructose diet
*YBX1*-KO^hep^: Hepatocyte-specific YBX1 knockout mice
LW/BW: liver-to-body weight ratio
PA: palmitic acid
