# Supplementary material for: YBX1/CD36 positive feedback loop-mediated lipid accumulation drives metabolic dysfunction-associated steatotic liver disease
Source: Int J Biol Sci. 2025 Feb 18;21(5):2118–34. doi: 10.7150/ijbs.105798 (PMC11900817; doi:10.7150/ijbs.105798)
Supplement: Supplementary file 1 — Supplementary materials and methods, figures. [file ijbsv21p2118s1.pdf]

YBX1/CD36 positive feedback loop-mediated lipid accumulation drives metabolic dysfunction-associated steatotic liver disease

Materials and methods

Histology, immunohistochemistry, and immunofluorescence

Standard Hematoxylin and Eosin (H&E) staining of slides was performed to assess the extent of steatosis. Frozen liver tissue samples were stained with oil red O. Lipid droplet dyes were stained with Bodipy493/503 (diluted 1:1000, Thermo Fisher) and Nile Red (MCE, HY-D0718). Liver tissue sections and cell slides were simultaneously stained for YBX1, CD36, HNF4α, and Bodipy using an advanced seven-color multiplex immunofluorescence kit (Recordbio Biological Technology, Shanghai, China). The staining protocol was optimized by incorporating tyramide signal amplification (TSA) technology, under strict adherence to the manufacturer's specified guidelines. Immunohistochemistry (IHC) was utilized to quantify the expression levels of YBX1 and CD36 in liver tissue sections. The primary antibodies used were anti-YBX1 (Abcam, ab76149), anti-HNF4α (Abcam, ab201460), and anti-CD36 (Abcam, ab255331), diluted at ratios of 1:100, 1:8000, and 1:800, respectively. A polyclonal HRP-conjugated secondary antibody was diluted at a ratio of 1:1000. Fluorescence imaging was conducted using the STELLARIS 8 DIVE (Leica).

Biochemical measurements

Plasma levels of triglycerides (TGs), total cholesterol (TC), alanine aminotransferase (ALT), and aspartate aminotransferase (AST) were quantified following the manufacturer's specified protocols (Leidu Company, Chemray 240). To determine the hepatic TG content, approximately 50 mg of liver tissue was homogenized in 1 mL of 5% NP-40 solution, heated in a 95 °C water bath for 2–5 min, and subsequently centrifuged at 15,000*g* for 10 min. The resulting supernatants were collected and analyzed using a triglyceride quantification kit (Wako, 632-50991).

Western blotting

Cells and tissues were lysed on ice for 30 min using radio immunoprecipitation assay buffer (RIPA) supplemented with a protease and phosphatase inhibitor cocktail (Beyotime Biotechnology, P1045). Protein concentrations were subsequently quantified using the Enhanced BCA Protein Assay Kit (Beyotime Biotechnology, P0009). After quantification, the proteins were separated by SDS-PAGE and subjected to immunoblotting with antibodies specific for YBX1 (CST, D299), phosphorylated YBX1 (CST, C34A2), CD36 (Abcam, ab255331), AKT (Abclone, A18675), phosphorylated AKT (Abclone, Ap0637), and β-actin (Abcam, ab8226). Protein detection was carried out by utilizing the ECL Chemiluminescent Kit (Thermo Fisher Scientific, 32209). Images were captured with the Amersham Imager 600 system.

Quantitative RT-PCR and RNA sequencing

Total RNA was isolated from liver tissues or cells utilizing the Trizol reagent (Invitrogen, 15596–026). Subsequent reverse transcription was conducted using the PrimeScript RT reagent kit (TakaRa, RR047A). Quantitative real-time PCR (qPCR) was then performed using the One Step TB Green® PrimeScript™ RT-PCR Kit (TakaRa, RR066A). Data analysis was executed according to the $2^{-\Delta\Delta Ct}$ method, with β-actin serving as the internal control. The following sequences of the primers were employed: CD36 (F 5'-GGACATTGAGATTCTTTTCCTCTG-3 and 5'-GCAAAGGCATTGGCTGGAAGAAC-3'); YBX1 (F 5'-CAGCAGACCGTAACCATTATAGAC-3 and 5'-CTCGTTCTTTTCCCCACTCTC-3'). RNA sequencing libraries were prepared and sequenced by Novogene Co., Ltd. The resulting data were analyzed using the NovoMagic online analytical tool.

Chromatin immunoprecipitation

The chromatin immunoprecipitation (ChIP) was performed utilizing a ChIP kit (Thermo Fisher Scientific, Cat: 26162) in accordance with the provided protocol. In brief, AML12 cells were subjected to crosslinking with 1% formaldehyde for 10 min at 37 °C, followed by treatment with 125 mM glycine for 5 min. Subsequently, the cells were lysed in a sodium dodecyl sulfate (SDS) buffer containing 1 mM phenylmethylsulfonyl fluoride (PMSF). The crosslinked

chromatin was fragmented by sonication. Precleared chromatin samples were subjected to overnight immunoprecipitation at 4 °C using anti-YBX1 pAb (MBL, RN015P) or β-actin. The precleared samples not treated with primary antibodies were employed as the input. The samples were then used as templates in qPCR for the quantitative detection of the binding motif of YBX1 within the promoter of CD36, as predicted by the JASPAR database. The following primer sequences were used in qPCR: CD36 S1 (F 5'-TGACCAAGCATCTGGCAAGT-3 and R 5'-CTAAGGGGAGGAGAGGGCAT-3'); CD36 S2 (F 5'-TGATGGCTAGCCTGCAACAA-3 and 5'-GCATTTTGCCGTGTGTGAGT-3').

Dual luciferase reporter assays

The promoter region of CD36 (–2000 bp to +100 bp from the transcription start site of CD36) was cloned into the pGL3−promoter vector to generate the reconstructed plasmid pGL3−CD36 promoter. The binding motif of YBX1 was deleted and introduced into the pGL3-CD36 promoter to generate the mutant pGL3-CD36 Mut. Full length of the *YBX1* gene was constructed using pcDNA 3.1 vectors. The YBX1-containing pcDNA 3.1 construct or the pcDNA 3.1 vector was transfected into HEK293T cells, along with pGL3-basic, pGL3-CD36 promoter, or pGL3-CD36 Mut, using lipofectamine 3000 (Invitrogen, L3000150). After 48 h of transfection, the HEK293T cells were lysed and the activity of firefly luciferase and Renilla luciferase in each well was quantified utilizing the Firefly & Renilla Light Luciferase Reporter Assay kit (Meilunbio, MA0520-1), in accordance with the manufacturer's protocol. The activity of firefly luciferase was normalized to that of Renilla luciferase.

Nucleocytoplasmic separation

Primary hepatocytes were isolated from the mice fed on a chow diet or HFCFD. They were plated and collected after 12 h of culture. The AML12 cells were treated with bovine serum albumin (BSA) or PA for 24 h before harvesting. Nucleocytoplasmic separation was carried out using the nuclear and cytoplasmic protein extraction kit (Thermo Fisher Scientific, 78835), in accordance with the manufacturer's protocol. Briefly, disruption of cell membrane and release of cytoplasmic protein were performed by adding buffer CER I and II to AML12 cells. After centrifugation, cytoplasmic protein and intact nuclei were obtained from the supernatant and

pellet, respectively. Then, buffer NER was added to the pellet to extract nuclei protein.

Flow cytometry analysis

Flow cytometry was employed to assess apoptosis in AML12 cells treated with PA (with the concentration varying from 0 to 500 μM) for 24 h. The cells were resuspended in the binding buffer and subsequently labeled with Annexin V and propidium iodide (PI) (Beyotime, C1062S), as per the manufacturer's protocol for apoptosis evaluation.

Transmission electron microscopy

The primary hepatocytes were fixed in 2.5% glutaraldehyde dissolved in a phosphate buffer for 4 h at room temperature. Subsequently, the cells were washed four times with 0.1 M phosphate buffer for 10 min each and then fixed in 1% OsO_4 in 0.1 M phosphate buffer (pH 7.0) for 1.5 h. The samples underwent dehydration when the concentrations of ethanol and acetone were increased. They were then embedded in Epon 812 and polymerized in an oven for 48 h at 60 ℃. Ultra-thin sections (approximately 100-nm thick) were prepared using LEICA EM UC7 and subsequently double-stained with lead citrate, omitting uranium dye. Imaging was performed using a Thermo Fisher Tecnai G2 transmission electron microscope operating at 120 kV.

Seahorse XF assay

Cellular fatty acid oxidation was evaluated by utilizing the Seahorse XF Palmitate Oxidation Stress Test Kit (Agilent Technologies, 103693-100). The cells were cultured in XF-compatible plates and incubated in a substrate-limited medium overnight. The experiments were conducted using a Seahorse XF Analyzer to measure the oxygen consumption rates following the administration of palmitate–bovine serum albumin complexes and various inhibitors, including oligomycin, FCCP (carbonyl cyanide p-(trifluoromethoxy)phenylhydrazone), and rotenone/antimycin A. These measurements were adopted to evaluate both basal and maximal respiration. The data were normalized and analyzed using Seahorse XF Wave software.

Datasets and bioinformatic analysis

RNA sequencing data, including GSE193084, GSE135251, and GSE15653, were obtained

from the Gene Expression Omnibus (GEO) database (https://www.ncbi.nlm.nih.gov/geo/). The Limma 3.5 0.1 package was employed to analyze gene expression levels between the control and NAFLD groups. Genes with a fold change of 2.0 and a P-value < 0.05 were considered differentially expressed.

Human single-cell sequencing data (ScRNA-seq) of the GSE212837 dataset comprising 27,001 genes and 27,914 cells were downloaded from the GEO database. Cell clustering and annotation were performed using the "Seurat" R package [1]. The SCENIC algorithm was applied to predict the transcription factor in each cell type.

Mouse single-cell RNA sequencing data were obtained from the GSE129516 dataset, comprising 33,168 cells. Of these, 17,788 cells were derived from the chow-fed mice and the remaining 15,380 cells were obtained from the HFCFD-fed mice. The procedures for cell clustering and annotation are comprehensively described elsewhere [2]. The annotated data were visualized using Uniform Manifold Approximation and Projection (UMAP). The expression levels of the *YBX1* gene are depicted through a topographic map and a bubble chart.

References

[1] Hao Y, Hao S, Andersen-Nissen E, Mauck WM, Zheng S, Butler A, et al. Integrated analysis of multimodal single-cell data. Cell 2021;184:3573-3587.e29. https://doi.org/10.1016/j.cell.2021.04.048.

[2] Xiong X, Kuang H, Ansari S, Liu T, Gong J, Wang S, et al. Landscape of Intercellular Crosstalk in Healthy and NASH Liver Revealed by Single-Cell Secretome Gene Analysis. Mol Cell 2019;75:644-660.e5. https://doi.org/10.1016/j.molcel.2019.07.028.

Supplementary Table

Table S1 and Table S1 are detailed in Excel spreadsheets 1 and 2, respectively, provided in the Supplementary Material.

Table S3. Antibodies

Name	Supplier	Cat no.
Bodipy493/503	Thermo Fisher	D2191
Nile Red	MCE	HY-D0718
YBX1	Abcam	ab76149

CD36	Abcam	ab255331
HRP-mouse IgG	Cell Signaling Technology	7076S
YBX1	Cell Signaling Technology	D299
β-actin	Abcam	ab8226
HNF4α	Abcam	ab201460
Phosphorylated YBX1	CST	C34A2
AKT	Abclone	A18675
Phosphorylated AKT	Abclone	Ap0637

Supplementary Figures and Figure Legends

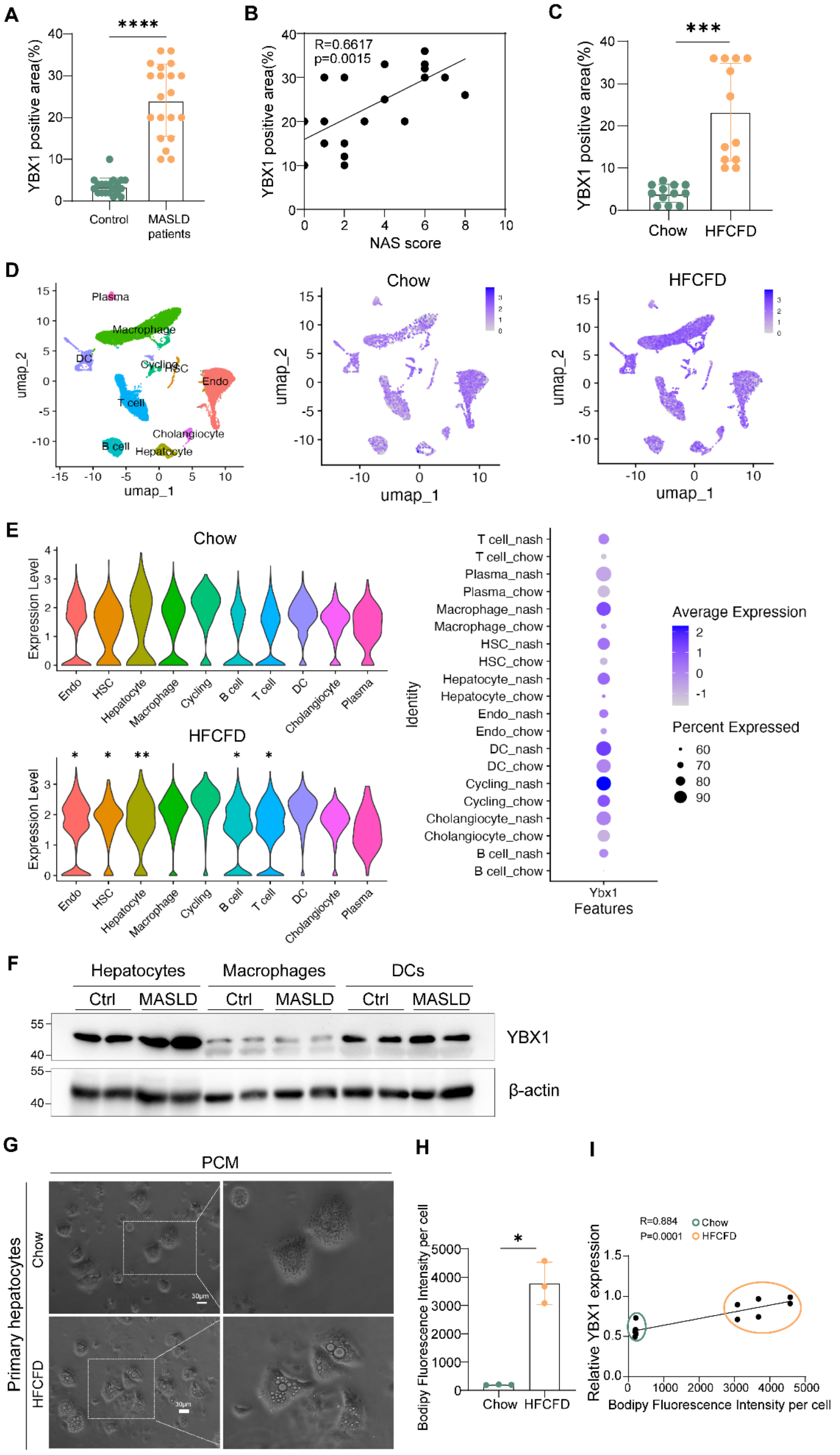

Figure S1, related to Figure 1. YBX1 Upregulation in MASLD: Mouse, Human, and Single-Cell RNA sequencing data.

A. Comparison of YBX1 score between MASLD and control group (control group, n = 10; MASLD group, n =10).

B. Correlation analysis of YBX1 IHC score and NAS score from MASLD and control group (control group, n = 10; MASLD group, n =10), R=0.788, $p < 0.0001$.

C. Comparison of YBX1 score between mice fed with Chow and HFCFD group, n=6 mice per group.

D. UMAP visualization of cellular annotations derived from mouse single cell sequencing data.

E. Representation of YBX1 gene expression in mouse single cell sequencing data using violin and bubble diagram.

F. Western blotting analysis of YBX1 expression of primary hepatocyte, macrophages and DCs isolated from mice fed with Chow or HFCFD, n=2 mice per group.

G. Representative images of primary hepatocytes isolated from mice fed with Chow or HFCFD visualized using Phase Contrast Microscopy. (Scale bar, 30μm).

H. Comparison of bodipy fluorescence intensity per cell between Chow diet and HFCFD group (n=3 mice per group).

I. Correlation analysis of YBX1 protein expression and bodipy fluorescence intensity per cell, n=6 mice per group. R=0.884, p=0.0001.

Data are presented as mean ± SD, with biologically individual data points shown. *$p < 0.05$, **$p < 0.01$, ***$p < 0.001$, ****$p < 0.0001$. P values were determined by unpaired two-tailed Student's t-test with Welch's correction (A, C, H) and linear correlation and regression analyses (B, I). HFCFD, high-fat-cholesterol and high-fructose diet; PCM, Phase Contrast Microscope.

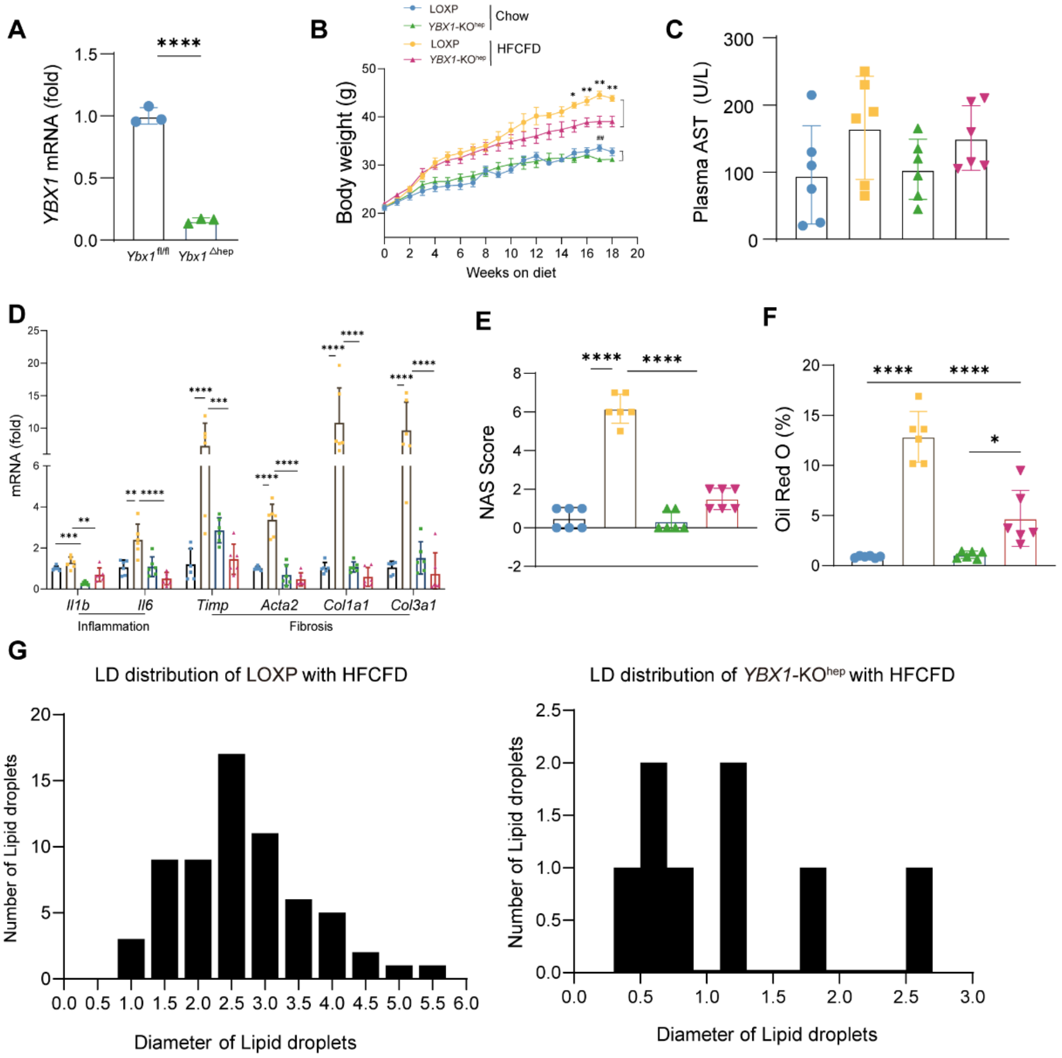

A
B
C
D
E
F
G
YBX1 mRNA (fold)
Ybx1fl/fl
Ybx1Δhep
LOXP
YBX1-KOhep
Chow
HFCFD
Body weight (g)
Weeks on diet
Plasma AST (U/L)
mRNA (fold)
Il1b
Il6
Timp
Acta2
Col1a1
Col3a1
Inflammation
Fibrosis
NAS Score
Oil Red O (%)
LD distribution of LOXP with HFCFD
LD distribution of YBX1-KOhep with HFCFD
Number of Lipid droplets
Diameter of Lipid droplets

Figure S2, related to Figure 2. Hepatocyte-specific *YBX1* deletion alleviates hepatic steatosis.

A. RT-qPCR analysis of *YBX1* expression in the primary hepatocytes isolated from Loxp and *YBX1*-KO^{hep} mice (n=3 mice per group).

B. Body weight change of Loxp and *YBX1*-KO^{hep} mice fed with Chow or HFCFD (n=6 mice per group).

C. Plasma levels of AST in Loxp and *YBX1*-KO^{hep} mice fed with Chow or HFCFD (n=6 mice per group).

D. Relative mRNA levels of inflammation and fibrosis-associated genes in the liver of Loxp and *YBX1*-KO^{hep} mice fed with Chow or HFCFD. n = 6 per group.

E. NAS Score in Loxp and *YBX1*-KO^{hep} mice fed with Chow or HFCFD (n=6 mice per group).

F. Quantification of Oil Red O in Loxp and *YBX1*-KO^{hep} mice fed with Chow or HFCFD (n=6 mice per group).

G. Lipid droplets distribution in Loxp and *YBX1*-KO^{hep} mice fed with HFCFD.

Data are presented as mean ± SD, with biologically individual data points shown. *p < 0.05, **p < 0.01, ***p < 0.001, ****p < 0.0001. P values were determined by unpaired two-tailed Student's t-test (A), pairwise t-test (B) and one-way ANOVA followed by Tukey's test (C-F). HFCFD, high-fat-cholesterol and high-fructose diet; LD, lipid droplet; *YBX1*-KO^{hep}, hepatocyte-specific *YBX1*-deficient mice.

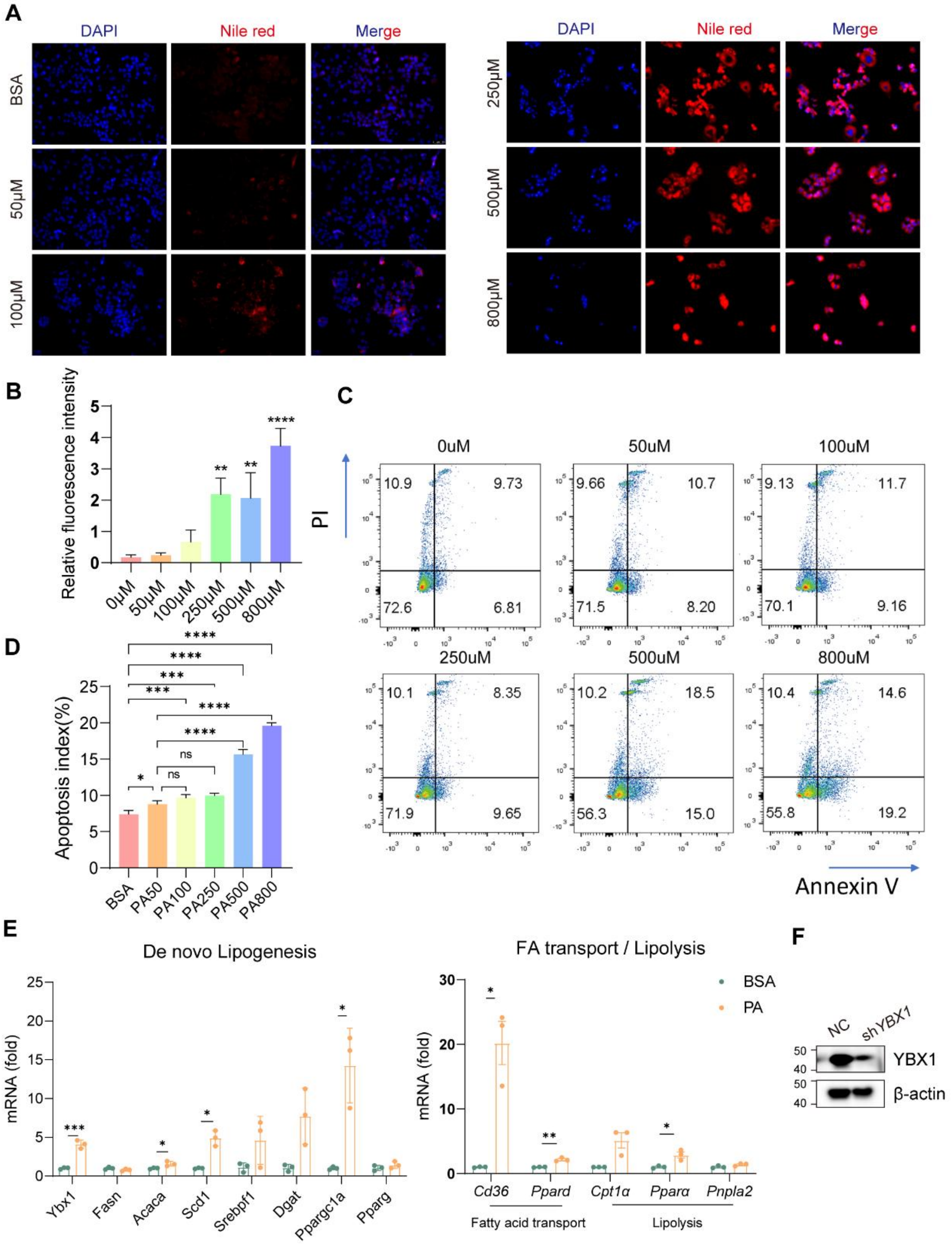
A
DAPI
Nile red
Merge
BSA
50μM
100μM
250μM
500μM
800μM
B
Relative fluorescence intensity
0uM
50uM
100uM
250uM
500uM
800uM
C
0uM
50uM
100uM
250uM
500uM
800uM
PI
Annexin V
10.9
9.73
72.6
6.81
9.66
10.7
71.5
8.20
9.13
11.7
70.1
9.16
10.1
8.35
71.9
9.65
10.2
18.5
56.3
15.0
10.4
14.6
55.8
19.2
D
Apoptosis index(%)
BSA
PA50
PA100
PA250
PA500
PA800
ns
E
De novo Lipogenesis
mRNA (fold)
Ybx1
Fasn
Acaca
Scd1
Srebpf1
Dgat
Ppargc1a
Pparg
FA transport / Lipolysis
BSA
PA
Cd36
Ppard
Cpt1α
Pparα
Pnpla2
Fatty acid transport
Lipolysis
F
NC
shYBX1
YBX1
β-actin

Figure S3, related to Figure 3. Establishment of AML12 lipid deposition model and validation of the efficacy of stable sh*YBX1* cell line.

A. Representative images of Nile red (red) staining of AML12 treated with PA at concentrations varying from 50μM to 800μM.

B. Quantification of Nile red staining of AML12 treated with PA at concentrations varying from 50μM to 800μM (n = 3 biologically independent experiments).

C. Assessment of Apoptosis in AML12 cell treated with 50μM to 800μM concentrations of PA by Flow Cytometry

D. Quantification of Apoptosis in AML12 cell treated with PA at concentrations varying from 50μM to 800μM (n = 3 biologically independent experiments).

E. Relative mRNA levels of genes related to de novo lipogenesis, fatty acid transport and lipolysis in AML12 cell treated with 250μM PA or solvent (n = 3 biologically independent experiments).

F. Western blotting analysis of YBX1 expression in NC and sh*YBX1* cells.

Data are presented as mean ± SD, with biologically individual data points shown. *p < 0.05, **p < 0.01, ***p < 0.001, ****p < 0.0001. P values were determined by unpaired two-tailed Student's t-test (YBX1, Fasn, Acaca, Srebpf1, Pparg, Ppard, Ppara and Pnpla2 group of E), unpaired two-tailed Student's t-test with Welch's correction (Scd1, Dgat, Ppargc1a, CD36 and Cpt1a group of E) and one-way ANOVA followed by Tukey's test (B, D). PA, palmitic acid; sh*YBX1* cells, cells with YBX1 stable knocked down by short hairpin RNA.

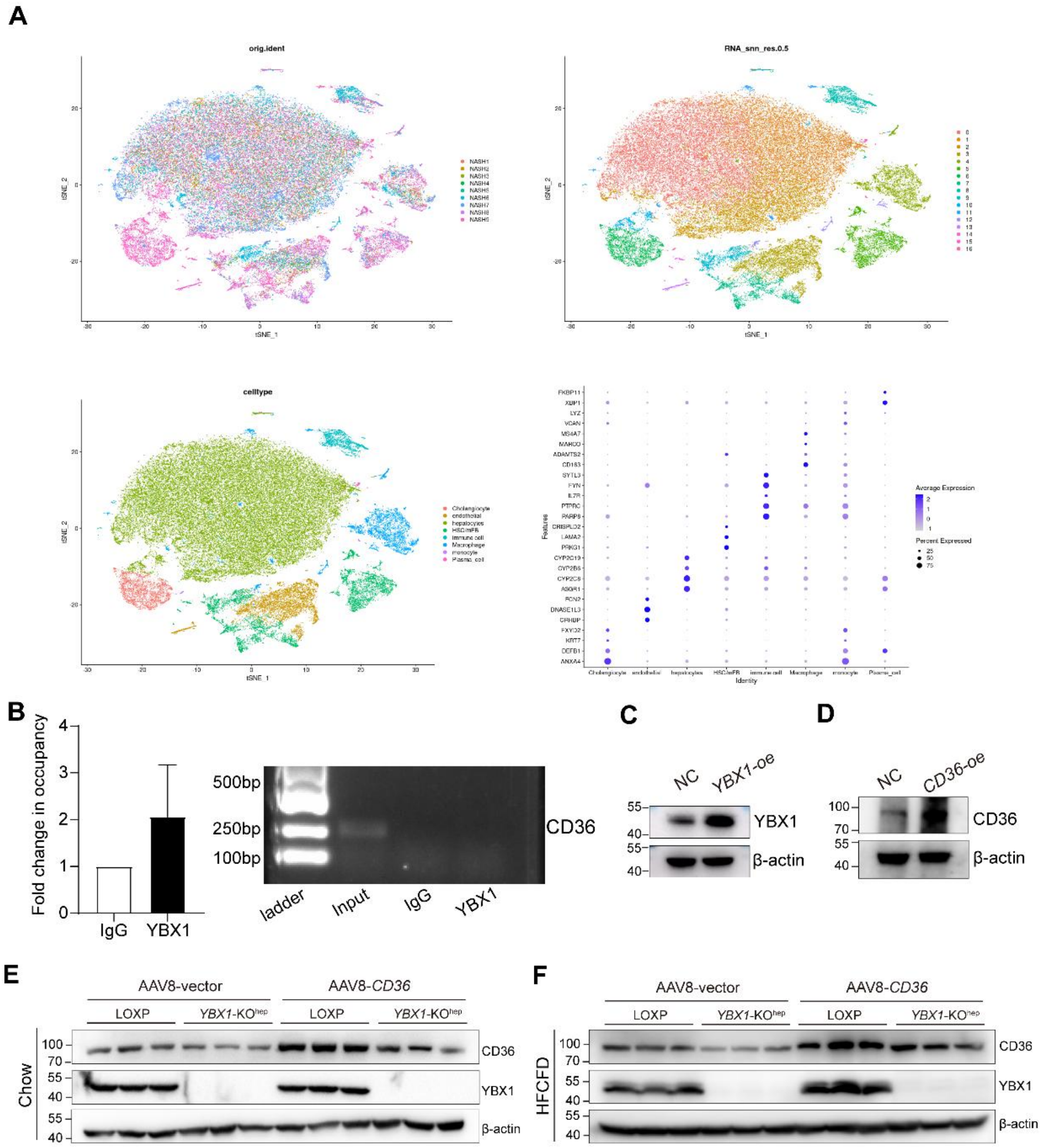
A
orig.ident
RNA_snn_res.0.5
celltype
B
Fold change in occupancy
IgG
YBX1
500bp
250bp
100bp
CD36
ladder
Input
IgG
YBX1
C
NC
YBX1-oe
YBX1
β-actin
D
NC
CD36-oe
CD36
β-actin
E
AAV8-vector
AAV8-CD36
LOXP
YBX1-KOhep
LOXP
YBX1-KOhep
Chow
CD36
YBX1
β-actin
F
AAV8-vector
AAV8-CD36
LOXP
YBX1-KOhep
LOXP
YBX1-KOhep
HFCFD
CD36
YBX1
β-actin

Figure S4, related to Figure 4, Figure 6 and Figure 7. YBX1 facilitates lipid accumulation in hepatocytes by upregulating CD36.

A. Clustering of cell types in the liver in single-cell transcriptomic profile of GSE212837 dataset.

B. CHIP-qPCR assay and DNA banding images of ChIP product of F2 fragments of YBX1(n = 3 biologically independent experiments).

C. Western blotting analysis of YBX1 expression in AML12 cells transfected with YBX1 overexpression plasmids or control.

D. Western blotting analysis of CD36 expression in AML12 cells transfected with CD36 overexpression plasmids or control.

E. Western blotting analysis of YBX1 and CD36 expression in Loxp mice and *YBX1*-KO^{hep} mice with or without AAV8-*CD36* injection in chow group (n=3 mice per group).

F. Western blotting analysis of YBX1 and CD36 expression in Loxp mice and *YBX1*-KO^{hep} mice with or without AAV8-*CD36* injection in HFCFD group (n=3 mice per group).

Data are presented as mean ± SD, with biologically individual data points shown. *$p < 0.05$, **$p < 0.01$, ***$p < 0.001$, ****$p < 0.0001$. P values were determined by unpaired two-tailed Student's t-test with Welch's correction (B). YBX1-oe, pCDH plasmids overexpressing YBX1; CD36-oe, pCDH plasmids overexpressing CD36; AAV8-*CD36*, adeno-associated virus-mediated overexpression of CD36.
